# STAT3 ameliorates cognitive deficits by positively regulating the expression of NMDARs in a mouse model of FTDP-17

**DOI:** 10.1038/s41392-020-00290-9

**Published:** 2020-12-26

**Authors:** Xiao-Yue Hong, Hua-Li Wan, Ting Li, Bing-Ge Zhang, Xiao-Guang Li, Xin Wang, Xiao Li, Qian Liu, Chong-Yang Chen, Ying Yang, Qun Wang, Shu-Peng Li, Hao Yu, Jian-Zhi Wang, Xi-Fei Yang, Gong-Ping Liu

**Affiliations:** 1 Department of Pathophysiology, School of Basic Medicine and the Collaborative Innovation Center for Brain Science, Key Laboratory of Ministry of Education of China and Hubei Province for Neurological Disorders, Tongji Medical College, Huazhong University of Science and Technology, Wuhan, 430030 China; 2 Clinic Center of Human Gene Research, Union Hospital, Tongji Medical College, Huazhong University of Science and Technology, Wuhan, China; 3 State Key Laboratory of Oncogenomics, School of Chemical Biology and Biotechnology, Peking University Shenzhen Graduate School, Shenzhen, China; 4 Bomedical Engineering, Shenzhen Polytechnic, Shenzhen, 518055 China; 5 Key Laboratory of Modern Toxicology of Shenzhen, Shenzhen Center for Disease Control and Prevention, Shenzhen, 518055 China; 6 Co-innovation Center of Neuroregeneration, Nantong University, Nantong, Jiangsu 226001 China

**Keywords:** Diseases of the nervous system, Molecular neuroscience

## Abstract

In tauopathies, memory impairment positively strongly correlates with the amount of abnormal tau aggregates; however, how tau accumulation induces synapse impairment is unclear. Recently, we found that human tau accumulation activated Signal Transduction and Activator of Transcription-1 (STAT1) to inhibit the transcription of synaptic N-methyl-D-aspartate receptors (NMDARs). Here, overexpressing human P301L mutant tau (P301L-hTau) increased the phosphorylated level of Signal Transduction and Activator of Transcription-3 (STAT3) at Tyr705 by JAK2, which would promote STAT3 translocate into the nucleus and activate STAT3. However, STAT3 was found mainly located in the cytoplasm. Further study found that P301L-htau acetylated STAT1 to bind with STAT3 in the cytoplasm, and thus inhibited the nuclear translocation and inactivation of STAT3. Knockdown of STAT3 in STAT3^flox/flox^ mice mimicked P301L-hTau-induced suppression of NMDARs expression, synaptic and memory impairments. Overexpressing STAT3 rescued P301L-hTau-induced synaptic and cognitive deficits by increasing NMDARs expression. Further study proved that STAT3 positively regulated NMDARs transcription through direct binding to the specific GAS element of NMDARs promoters. These findings indicate that accumulated P301L-hTau inactivating STAT3 to suppress NMDARs expression, revealed a novel mechanism for tau-associated synapse and cognition deficits, and STAT3 will hopefully serve as a potential pharmacological target for tauopathies treatment.

## Introduction

Tau pathology, composed of the misfolded hyperphosphorylated Tau,^1^ is involved in more than 20 kinds of neurodegenerative diseases, including Alzheimer’s disease (AD), frontotemporal dementia (FTDP-17), progressive supranuclear palsy, and so on, which collectively referred to as “tauopathies”.^2–4^ Braak staging is used to describe the stages and location of neurofibrillary tangles in AD,^5^ and the degree of neurodegeneration and memory impairment are strongly correlated with the amount of abnormal tau aggregates in the brain^6^. In addition, the total level of tau protein in cerebrospinal fluid is inversely related to score memory.^6,7^

Animal studies strongly support clinical evidence that tau plays a key role in learning and memory disorders. For example, transgenic mice expressing the most common FTDP-17 mutation (P301L) develop neurofibrillary tangles and behavioral deficits in an age- and gene-dose-dependent manner.^8^ Overexpression of full-length human tau40 (htau) also triggers pathological changes and memory impairment in the brain of transgenic mice,^9,10^ while inhibition of tau expression or immunotherapy against pathological tau improves memory impairment and reduces neuronal loss in htau transgenic mice.^11,12^

Tau protein is essential for the neurotoxic effects of Aβ, another key pathological component of AD. Knocking-out tau protein or truncated tau (Deltatau) can improve memory deficits found in Aβ-forming APP23 transgenic mice overexpressing human APP with the Swedish double mutation,^13^ and reducing endogenous tau can improve behavioral disorders of transgenic mice overexpressing human APP, but does not alter Aβ levels.^14^ Phosphorylation of tau at specific sites can also inhibit Aβ neurotoxicity.^15^ Potential of passive immunization targeting proximal N-terminal domain tau 6–18 inhibits not only tau but also Aβ pathology.^16^

Tau is a key microtubule-associated protein that promotes the assembly and maintains the stability of microtubules in nerve cells required for axonal transport and maintenance of neuronal integrity.^17,18^ Tau hyperphosphorylation results in the dissociation of microtubules and disruption of molecular axonal transport. Recent studies show that phosphorylated tau supports cell viability by antagonizing apoptotic factors.^19–21^ Tau hyperphosphorylation occurs not only in axons but also in cell bodies and dendrites.^13^

We have reported elsewhere that abnormal tau protein accumulates in cells in disorders of mitochondrial dynamics, mitophagy deficits, and mitochondria dysfunction associated with increasing mitochondrial membrane potential.^22,23^ In primary culture neurons, accumulated tau was found to activate calcineurin to dephosphorylate CREB and calcium/calmodulin-dependent protein kinase IV, which thereby perturbed intracellular calcium signaling.^24^ Tau accumulation also repressed autophagy by disrupting IST1-regulated ESCRT-III complex formation.^25^ These studies partially disclose the mechanisms underlying the toxic effects of tau. However, the molecular mechanisms underlying hTau-induced synapse impairment are not fully understood.

Recently, we found that accumulating htau increases JAK2 activity to phosphorylate STAT1, which decreased *N*-methyl-D-aspartate receptor (NMDAR) expression by directly binding to the specific GAS elements in NMDARs promoters (GluN1, GluN2A, GluN2B) and thereby inhibiting their transcription.^26^ STAT1 and STAT3, both belonging to STAT protein family, have similar specific DNA-binding element GAS and are both reported to involve in cognitive deficits induced by Abeta.^27–34^ In the present study, we found that overexpressing P301L-hTau (P301L), that the mutation can directly result in neurodegeneration,^35^ inhibited STAT3 to transport from the cytoplasm into the nucleus to inactivate STAT3 through increasing the interaction of acetylated STAT1 and STAT3 in the cytoplasmic fraction. Overexpressing STAT3 attenuated the P301L-induced synaptic and cognitive deficits. We also found that STAT3 can directly bind the specific GAS element in GluN1, GluN2A, or GluN2B promoter and thus activate expression of NMDARs, which reveals a novel mechanism underlying Tau-induced synapse impairment of cognitive deficits.

## Results

### Intracellular P301L-hTau accumulation inactivates STAT3 despite the level of phosphorylated STAT3 increases

In our previous study, we found that the accumulated htau increased STAT1 activity, while the activity of HNF1, HOX4C, PLAG1, SMUC, VDR, SF-1, and PIT1 decreased remarkably.^26^ STAT3 and STAT1 belong to the STAT protein family, and both are reported to play a role in cognitive deficits induced by Abeta.^27–34^ Herein, we investigated the effects of P301L-hTau accumulation on STAT3 activity, and if so, the role of STAT3 in P301L-hTau-induced cognitive deficits and the underlying molecular mechanisms.

**Fig. 1:**
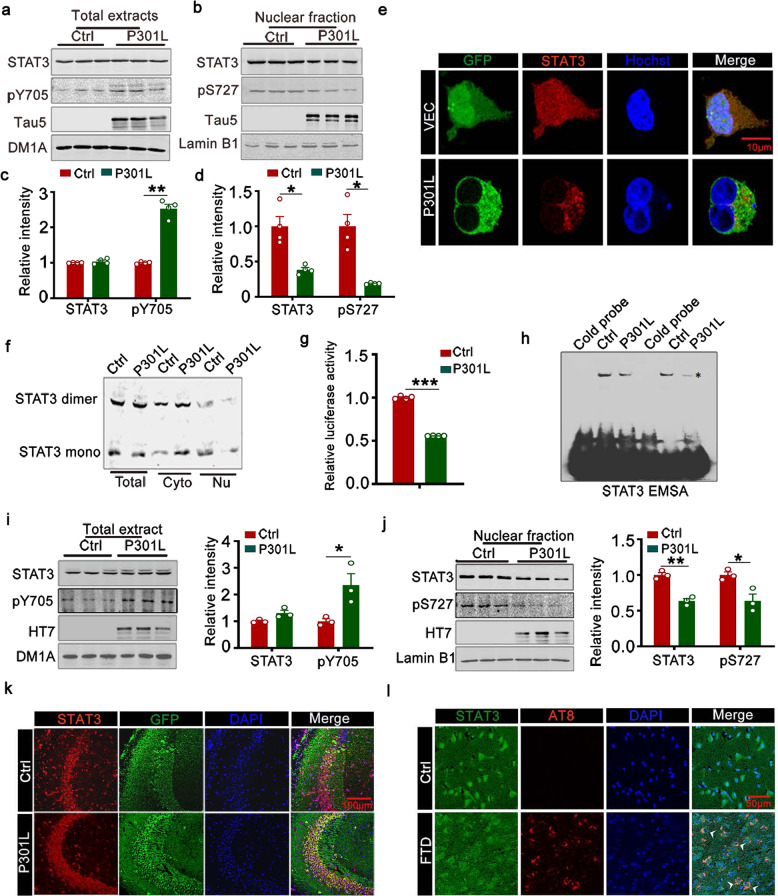
Intracellular P301L-hTau accumulation inactivates STAT3 via inhibition of nuclear translocation. Overexpression of human tau containing the most common FTDP-17 mutation (P301L-hTau, or P301L) increased phosphorylated STAT3 at Tyr705 (pY705) in whole-cell extracts (**a**) and decreased total STAT3 and STAT3 phosphorylated at Ser727 (pS727) in the nuclear fraction (**b**) detected by Western blotting and quantitative analysis (**c**, **d**). Two-tailed Student’s *t* test, **c** [pY705] *p* = 0.006; **d** [STAT3] *p* = 0.021; [pS727] *p* = 0.02, *N* = 4. The empty vector was transfected as a control (Ctrl). **e** The representative immunofluorescent images of STAT3 in HEK293 cells. **f** Overexpressing P301L significantly decreased STAT3 monomer and dimer formation in nuclear fraction (Nu), as determined by Western blotting. Cyto: cytoplasmic fraction. **g** Overexpressing P301L decreased STAT3 transcriptional activity relative to the vector control in HEK293 cells detected by luciferase assay. Two-tailed Student’s *t* test, *p* < 0.001, *n* = 4. **h** Overexpressing P301L decreased STAT3-DNA-binding activity in HEK293 cells measured by electrophoretic mobility shift assay (EMSA). * indicates STAT3/DNA complex. **i**, **j** AAV-P301L-hTau-eGFP (AAV-P301L) or the empty vector AAV-eGFP was stereotaxically injected into hipocampal CA3 of 2-month-old C57 mice for 1 month. The increased phosphorylated level of STAT3 (pY705) in hippocampal total extracts (**i**) and decreased total and phosphorylated STAT3 (pS727) in the nuclear fraction (**j**) in P301L group were measured by Western blotting and quantitative analysis. Two-tailed Student’s *t* test, **i** [pY705] *p* = 0.037; **j** [STAT3] *p* = 0.002; [pS727] *p* = 0.024, *n* = 3. **k** The representative immunofluorescent images of STAT3 in hippocampal CA3 region. **l** The representative immunofluorescent images of STAT3 in FTDP-17 patients. Arrowhead indicated the staining of decreased STAT3 in the nucleus. Data were presented as mean ± SEM

As a critical intermediate segment of the hippocampal trisynaptic circuit, CA3 receives signals from dentate gyrus through mossy fibers and the CA3 information processing can modulate activity in CA1 subset. The hippocampal CA3 region has been proposed as the key structure for the storage of spatial memory. In previous studies, we invariably observe that tau pathology appears most apparent and early in CA3 compared with the other regions in htau mice or the rats exposed to calyculin A or advanced glycation end products, suggesting that hippocampal CA3 may be more vulnerable to tauopathies. Here, we chose to overexpress P301L-hTau in CA3. Compared with the control, total tau protein level (tau-5) increased to 2.5 times by AAV-P301L-hTau virus infection (Supplementary Fig. S1). We demonstrated that overexpressing P301L-hTau markedly increased the phosphorylation of STAT3 at Tyr705 (pY705) in cell extracts (Fig. 1a, c), but decreased total STAT3 and the activity-dependent phosphorylation of STAT3 at Ser727 (pS727) in the nuclear fraction (Fig. 1b, d) with a decreased nuclear translocation (Fig. 1e) and dimerization (Fig. 1f) of STAT3 measured by Western blotting and immunofluorescence imaging. Inactivation of STAT3 by overexpressing P301L-hTau was also confirmed by the TF luciferase assay (Fig. 1g). By the electrophoresis mobility shift assay (EMSA), using an oligonucleotide probe containing STAT3 binding site, we found that P301L-hTau accumulation decreased binding of STAT3 to DNA, and this association was disrupted by using cold probe (Fig. 1h). To verify the specificity of STAT3 inactivation, we studied the effects of TDP-43 and alpha-synuclein on STAT3 in HEK293 cells. The results showed that overexpression of TDP-43 or α-synuclein did not significantly alter the total and nuclear levels of STAT3 and p-STAT3 (Supplementary Fig. S2), indicating that STAT3 inactivation may be specific for P301L-hTau. These in vitro data indicated that intracellular P301L-hTau accumulation inhibited STAT3 activity.

To test the in vivo effects of P301L-hTau accumulation on STAT3, we first injected stereotaxically AAV-P301L-hTau into the mouse hippocampi and detected the alterations of STAT3 and pY-STAT3 after 1 month. Expression of P301L-hTau was confirmed by Western blotting (Fig. 1i), overexpression of P301L-hTau significantly increased total pY705-STAT3 in hippocampal extracts but decreased total and pS727-STAT3 in the nuclear fraction (Fig. 1i, j). A decreased nuclear translocation of STAT3 was also measured by immunofluorescence imaging (Fig. 1k). In the cortex of FTDP patients which had a P301L mutation of Tau, STAT3 in the nucleus was also significantly decreased (Fig. 1l). These data provide the in vivo and human evidence for the role of P301L-hTau accumulation in inhibiting STAT3 activity.

### Intracellular P301L-hTau accumulation increases the interaction of STAT1 and STAT3 in the cytoplasm by acetylation of STAT1

**Fig. 2:**
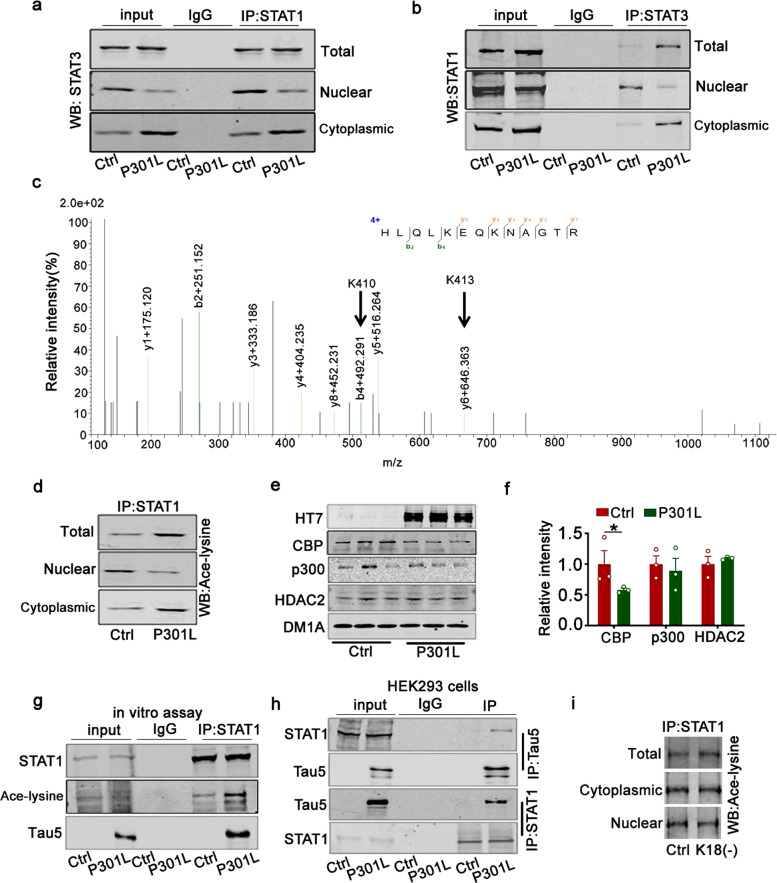
Overexpression of P301L-hTau increases acetylated level of STAT1 by tau acetyltransferase activity. **a**, **b** Overexpression of P301L increased the interaction of STAT1 and STAT3 in the cytoplasmic fraction of HEK293 cells measured by CO-IP. **c** Mass spectrometry analysis of the acetylated peptide of STAT1. The detected MS/MS peptide spectra are listed. **d** Overexpression of P301L increased the level of STAT1 acetylation in whole-cell extracts and the cytoplasm. Proteins were first immunoprecipitated with STAT1, followed by Western blotting with anti-ace-lysine antibody. **e**, **f** The CBP level decreased, and p300 and HDAC2 level remained unchanged while overexpressing P301L in HEK293 cells detected by Western blotting. Two-tailed Student’s *t* test, [CBP] *p* = 0.021, *n* = 3. **g** The P301L-hTau recombinant protein interacted with STAT1 and increased its acetylation level in the whole extracts of HEK293WT cells. **h** Tau interacted with STAT1 in HEK293WT cells transfected with P301L-hTau detected by the Co-IP assay. **i** P301L-K18(−) (P301L tau lacking the repeats) had no effect on acetylated STAT1 level. Data were presented as mean ± SEM

Phosphorylation of STAT3 at tyrosine 705 (pY-STAT3) is reported to lead to STAT3 nuclear translocation and DNA binding;^36,37^ however, in the present study, we found that P301L decreased the level of STAT3 in the nuclear fraction while pY-STAT3 in the total lysates increased. To probe for the cause, the Co-IP assay was used to detect the interaction of STAT3 and STAT1. This showed that intracellular P301L accumulation increased the interaction of STAT1 and STAT3 in both total lysates and the cytoplasm fraction but decreased the interaction in the nuclear fraction (Fig. 2a, b).

Previous studies reported that acetylation of STAT1 enhanced the binding of STAT1 with NF-κB p65 to inhibit NF-κB p65 nuclear translocation.^38^ To verify whether acetylation of STAT1 inhibited STAT3 nuclear translocation in the same way, we detected the acetylation of STAT1 by mass spectrometry (Fig. 2c). We obtained a lysine acetylation signal from the peptide fragment HLQLKEQKNAGTR, among which two lysine residues corresponded to lysine 410 (K410) and lysine 413 (K413) of STAT1, respectively. Intracellular P301L-hTau accumulation increased the acetylation of STAT1 in both cell lysates and the cytoplasm but decreased the acetylated level in the nuclear fraction by the IP assay (Fig. 2d).

To investigate the mechanisms underlying the increase of acetylated STAT1 induced by P301L-hTau, we measured CBP, p300, and HDAC2, which are involved in protein acetylation modification.^38–40^ However, P301L-hTau overexpression did not change the protein level of p300 and HDAC2 but decreased CBP in cell lysates (Fig. 2e, f), which together exclude involvement of CBP, p300, and HDAC2 in acetylating STAT1. Tau itself acts as acetyltransferase.^41^ In vitro assay showed that the recombinant protein P301L-hTau increased acetylated level of STAT1 (Fig. 2g). We also found that overexpressing P301L increased the binding with STAT1 in HEK293 cells (Fig. 2h). The repeat domain of tau protein, namely K18, mediates the acetyltransferase action of tau.^41^ We transfected the K18-deleted P301L (K18(−)) plasmid into HEK293 cells and found that K18(−) did not affect the acetylated STAT1 level and the interaction of STAT1 and STAT3 in total, cytoplasm and nuclear fraction compared with the control (Fig. 2i and Supplementary Fig. S3). The above results indicated that P301L-hTau accumulation inhibited STAT3 transport into the nucleus by increasing the interaction of STAT3 with STAT1 in the cytoplasm via acetylation of STAT1.

### STAT1 acetylation inhibited STAT3 nuclear translocation

**Fig. 3:**
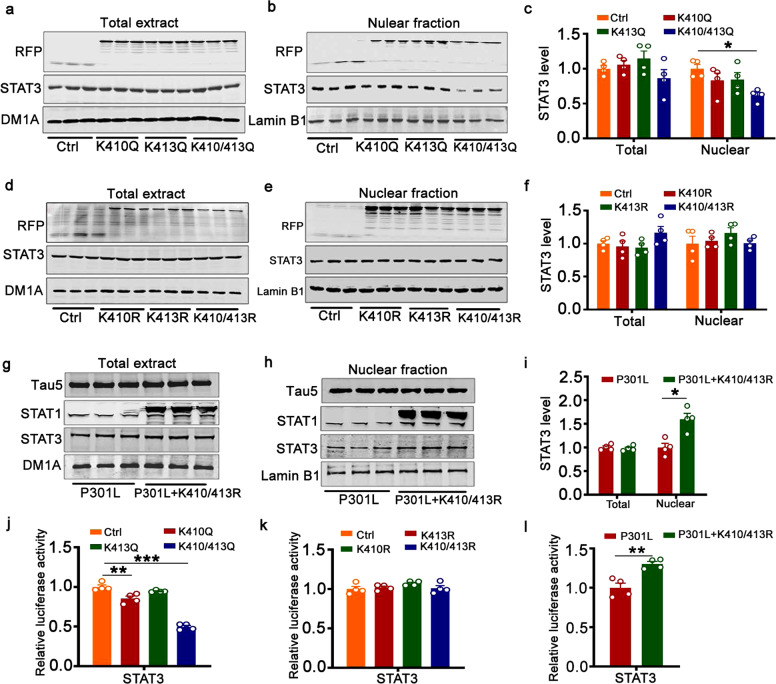
Acetylated STAT1 inhibited STAT3 nuclear translocation. Pseudoacetylated STAT1 mutant (K410/413Q) decreased total STAT3 in the nuclear fraction (**b**) but had no effect on the total STAT3 level in the whole extracts of HEK293 cells (**a**) detected by Western blotting and quantitative analysis (**c**). **c** One-way analysis of variance (ANOVA) followed by the Bonferroni’s post hoc test, nuclear STAT3 level [K410/413Q] *p* = 0.027, *n* = 4. The unacetylated (K410R, K413R, K410/413R) STAT1 mutant did not change STAT3 levels in total extracts (**d**) or the nuclear fraction (**e**), as determined by Western blotting and quantitative analysis (**f**). K410/K413R-STAT1 (K410/413R) restored P301L-induced reduction of decreased STAT3 in the nuclear fraction, as determined by Western blotting (**g**, **h**) and quantitative analysis (**i**). **i** Two-tailed Student’s *t* test, nuclear STAT3 level *p* = 0.044, *n* = 4. **j** K410/K413Q-STAT1 decreased the luciferase activity of STAT3. One-way analysis of variance (ANOVA) followed by the Bonferroni’s post hoc test, STAT3 luciferase activity [K410Q] *p* = 0.002; [K410/413Q] *p* < 0.001, *n* = 4. **k** Unacetylated (K410R, K413R, K410/413R) STAT1 mutant did not affect the luciferase activity of STAT3. **l** STAT1KR (K410/413R) rescued the decreased luciferase activity of STAT3 induced by P301L-hTau. Two-tailed Student’s *t* test, *p* = 0.007, *n* = 4. Data were presented as mean ± SEM

To further verify the effect of STAT1 acetylation on STAT3 nucleation, we used pseudoacetylated (K410Q, K413Q, and K410/413Q) or unacetylated (K410R, K413R, K410/413R) mutant STAT1 plasmids at single or double sites. Protein in the total cell lysis and nuclear fractions were extracted and detected by Western blotting, respectively. Only K410/413Q not K410Q or K413Q-STAT1 significantly reduced the level of STAT3 in the nuclear fraction, and the nuclear STAT3 level showed a decline with overexpression of pseudoacetylated mutant of STAT1 in single site (K410Q or K413Q) (Fig. 3a–c). Non-acetylated mutant STAT1 (K410R, K413R, or K410/413R) had no effect on the STAT3 level in the nuclear fraction (Fig. 3d–f). Neither pseudoacetylated nor unacetylated STAT1 mutant changed the STAT3 level in the total extract (Fig. 3a, c, d, f). The non-acetylated STAT1 mutant at the double site (K410/413R) ameliorated P301L-induced reduction of STAT3 level in the nuclear fraction (Fig. 3g–i). We also found by the luciferase assay that overexpression of K410/413Q-STAT1 plasmid decreased STAT3 activity, and K410/413R-STAT1 attenuated the P301L-induced reduced STAT3 activity (Fig. 3j–l).

### STAT3 knockdown by AAV-Cre induces synaptic plasticity and cognitive impairments in STAT3^flox/flox^ mice

**Fig. 4:**
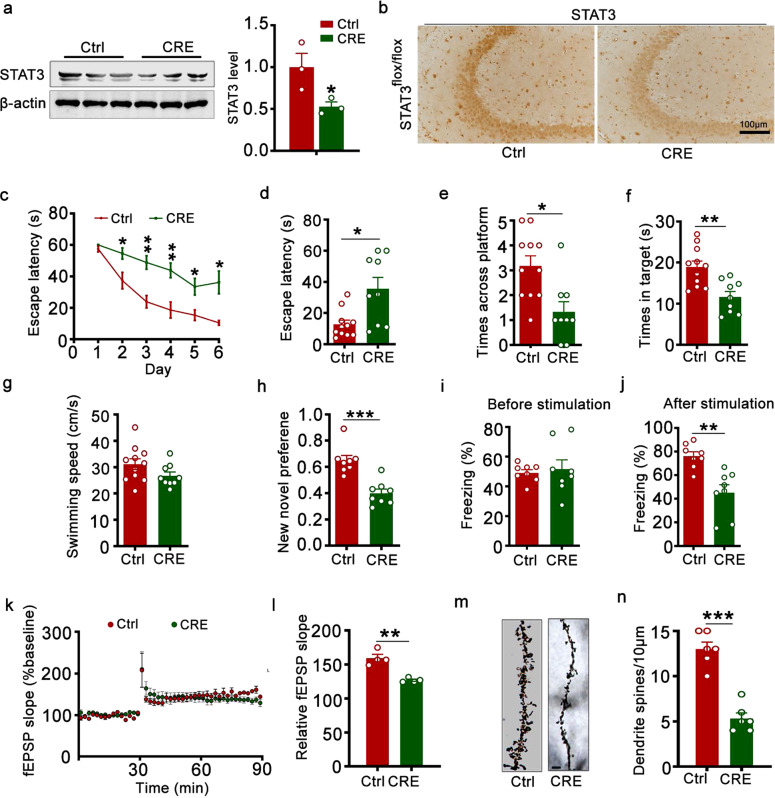
Downregulation of STAT3 induced synaptic impairments and memory deficits in STAT3^flox/flox^ mice. **a**, **b** AAV-Cre was stereotaxically injected into the hippocampal CA3 of 2-month-old STAT3^flox/flox^ mice. One month later, downregulation of STAT3 was confirmed by Western blotting (**a**) and immunohistochemical staining (**b**). Two-tailed Student’s *t* test, *p* = 0.031, *n* = 3. **c** STAT3 knockdown induced spatial learning deficits during Morris water maze (MWM) training. Two-way repeated measures analysis of variance (ANOVA) followed by the Bonferroni’s post hoc test, [day 2] *p* = 0.018, [day 3] *p* = 0.001, [day 4] *p* = 0.003, [day 5] p = 0.013, [day 6] *p* = 0.014, nCtrl/CRE = 11/9. STAT3 knockdown induced spatial memory deficits shown by the increased escape latency to reach the platform quadrant (**d**), the decreased crossing time in the platform site (**e**), and time spent in the target quadrant (**f**) measured at day 7 by removal of the platform in MWM test; no motor dysfunction was seen (**g**). Two-tailed Student’s *t* test; **d** *p* = 0.029; **e** *p* = 0.033; **f** *p* = 0.003; nCtrl/CRE = 11/9. **h** STAT3 knockdown induced cognition impairment shown by the decreased time spent in exploring the new novel object. Two-tailed Student’s *t* test, *p* < 0.001, *n* = 8. **i**, **j** STAT3 knockdown impaired long-term memory as shown by the decreased freezing time measured in the fear conditioning test. Two-tailed Student’s *t* test, **j** *p* = 0.0037, *n* = 8. STAT3 knockdown decreased slopes of field excitatory postsynaptic potentials (fEPSP (**k**)) recorded in hippocampal CA3, and quantitative analysis (**l**). Two-tailed Student’s *t* test, *p* = 0.0013, *n* = 7–8 slices from 4 mice per group. **m**, **n** STAT3 knockdown decreased the density of dendritic spines detected by Golgi staining. Scale bar, 5 μm. Two-tailed Student’s *t* test, *p* < 0.001, *n* = at least 22 neurons were analyzed from 6 mice per group. Data were presented as mean ± SEM

To investigate the role of STAT3 in learning and memory function,^32–34^ we first infused AAV-Cre into the hippocampal CA3 zone of 2-month-old STAT3^flox/flox^ mice to knockdown STAT3 and then assayed cognitive ability 1 month later (Fig. 4). The efficiency of AAV-Cre in downregulating STAT3 was confirmed by Western blotting (Fig. 4a) and immunohistochemistry (Fig. 4b). By MWM test, we observed that STAT3 knockdown efficiently induced learning impairments shown by the increase of escape latencies at days 2–6 during the 6-day training period (Fig. 4c). In the memory test (measured at day 7 by removal of the escape platform), STAT3 knockdown mice showed a longer average latency to reach the previous target quadrant (Fig. 4d); relative to control mice, they crossed the platform quadrant fewer times (Fig. 4e) and stayed for a shorter period in the platform quadrant (Fig. 4f). No significant difference in swimming speed was seen between the two groups (Fig. 4g), which excluded motor deficits. By the new object recognition test, STAT3 knockdown was associated with deficiency in recognizing a novel object (Fig. 4h). By contextual fear conditioning test, we also observed that STAT3 knockdown impaired long-term memory (LTM), as shown by a decreased freezing time during the memory test (Fig. 4j). The fEPSP slope and the density of dendrite spines were also reduced by STAT3 knockdown (Fig. 4k–n). These data demonstrate that downregulating STAT3 in the hippocampus can efficiently induce learning and memory impairments.

### STAT3 overexpression ameliorates P301L-hTau-induced synaptic plasticity and memory deficits

**Fig. 5:**
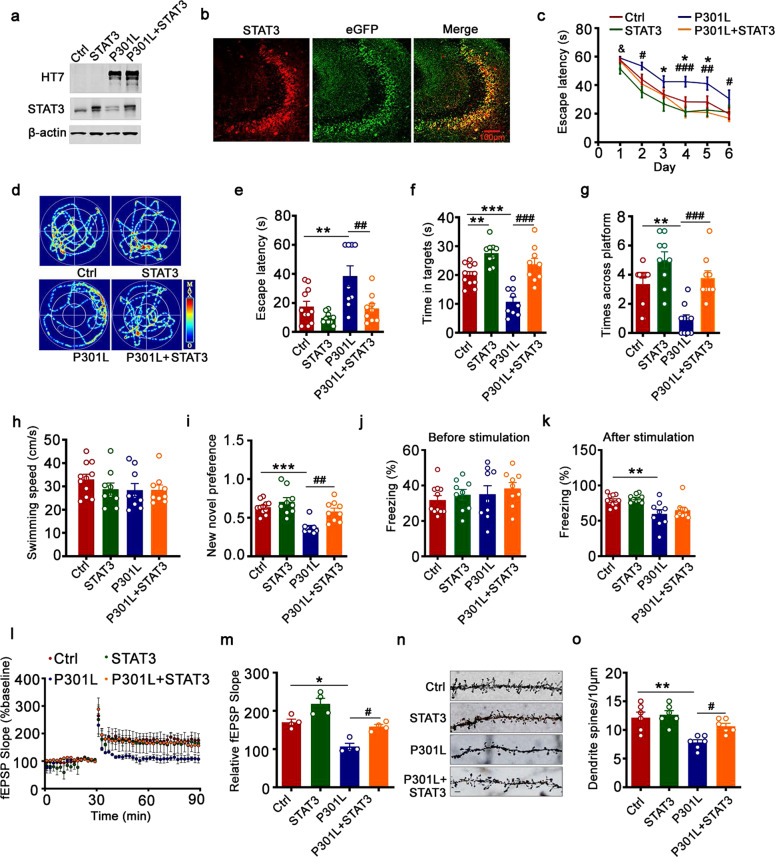
Overexpression of STAT3 ameliorates P301L-induced synaptic impairments and memory deficits. AAV-P301L-hTau-eGFP with or without AAV-STAT3 were stereotaxically injected into hippocampal CA3 of 2-month-old C57 mice. Learning and memory ability and synaptic plasticity were assessed 1 month later. Upregulation of STAT3 was confirmed by Western blotting (**a**) and immunofluorescent staining (**b**). **c** Upregulation of STAT3 ameliorated P301L-induced spatial learning deficits shown by the decreased escape latency during 6 consecutive days training in the Morris water maze (MWM) test. Two-way repeated measures analysis of variance (ANOVA) followed by the Bonferroni’s post hoc test, P301L vs. Ctrl [day 3] *p* = 0.013, [day 4] *p* = 0.034, [day 5] *p* = 0.003; STAT3 vs. Ctrl [day 1] *p* = 0.016; P301L + STAT3 vs. P301L [day 2] *p* = 0.032, [day 4] *p* < 0.001, [day 5] *p* = 0.007, [day 6] *p* = 0.043. NCtrl/STAT3/P301L/P301L + STAT3 = 11/9/9/9. Upregulation of STAT3 ameliorated hTau-induced spatial memory deficits as shown by the representative swimming trace (**d**), the decreased latency to reach the platform quadrant (**e**), the increased time spent in the target quadrant (**f**), and crossing times in the platform site (**g**) measured at day 7 by removing the platform in the MWM test; no motor dysfunction was seen (**h**). Two-way analysis of variance (ANOVA) followed by the Bonferroni’s post hoc test. **e** **p = 0.005, ##*p* = 0.005; **f** **p = 0.007, ***p < 0.001, ###*p* < 0.001; **g** **p = 0.003, ###*p* < 0.001. NCtrl/STAT3/P301L/P301L + STAT3 = 11/9/9/9. **i** Upregulation of STAT3 ameliorated P301L-induced cognitive impairment as shown by the increased time spent in exploring the new novel object measured at 24 h. Two-way analysis of variance (ANOVA) followed by the Bonferroni’s post hoc test. ***p < 0.001, ##*p* = 0.004; NCtrl/STAT3/P301L/P301L + STAT3 = 11/9/9/9. **j**, **k** Upregulation of STAT3 ameliorated P301L-induced contextual memory deficits measured at 24 h. Two-way analysis of variance (ANOVA) followed by the Bonferroni’s post hoc test. **p = 0.006; NCtrl/STAT3/P301L/P301L + STAT3 = 11/9/9/9. Overexpression of STAT3 restored slopes of field excitatory postsynaptic potentials (fEPSP (**l**)) recorded in hippocampal CA3, with quantitative analysis thereof (**m**). Two-way analysis of variance (ANOVA) followed by the Bonferroni’s post hoc test. *p = 0.03, #*p* = 0.012; *n* = 6–8 slices from 4 mice for each group. **n**, **o** Overexpression of STAT3 restored the density of dendritic spine detected by Golgi staining. Representative image (**n**) and quantitative analysis (**o**). Scale bar, 5 μm. Two-way analysis of variance (ANOVA) followed by the Bonferroni’s post hoc test. **p = 0.002, #*p* = 0.039; *n* = at least 22 neurons from 6 mice per group. Data were presented as mean ± SEM

To further certify the role of STAT3 in P301L-hTau-induced cognitive deficits, we co-injected AAV-P301L-hTau and AAV-STAT3 bilaterally into the hippocampal CA3 regions of 2-month-old C57 mice for 1 month. Overexpression of STAT3 was proved by Western blotting (Fig. 5a) and immunofluorescence (Fig. 5b). P301L-hTau impaired learning and memory abilities as detected by the MWM test (Fig. 5c–h), new novel object recognition test (Fig. 5i), and the conditional fear conditioning test (Fig. 5j, k), with reduction of fEPSP slope and dendrite spine density (Fig. 5l–o). Overexpression of STAT3 attenuated P301L-hTau-induced cognition and dendritic plasticity impairments (Fig. 5c–o). These data show that STAT3 plays a critical role in P301L-hTau-induced dendritic plasticity and cognitive deficits.

To explore whether STAT3 overexpression affects tau phosphorylation and aggregation, we extracted soluble and insoluble tau from the hippocampal CA3 and found that, overexpressing STAT3 reduced phosphorylated tau at Ser214, Thr231, Ser396, and Ser404 in the insoluble fraction (Supplementary Fig. S4). These data suggested that overexpressing STAT3 attenuated P301L-hTau-toxicity by reducing tau hyperphosphorylation and its pathological aggregation.

### STAT3 activates the transcription of NMDAR via binding to the specific GAS promoter element

**Fig. 6:**
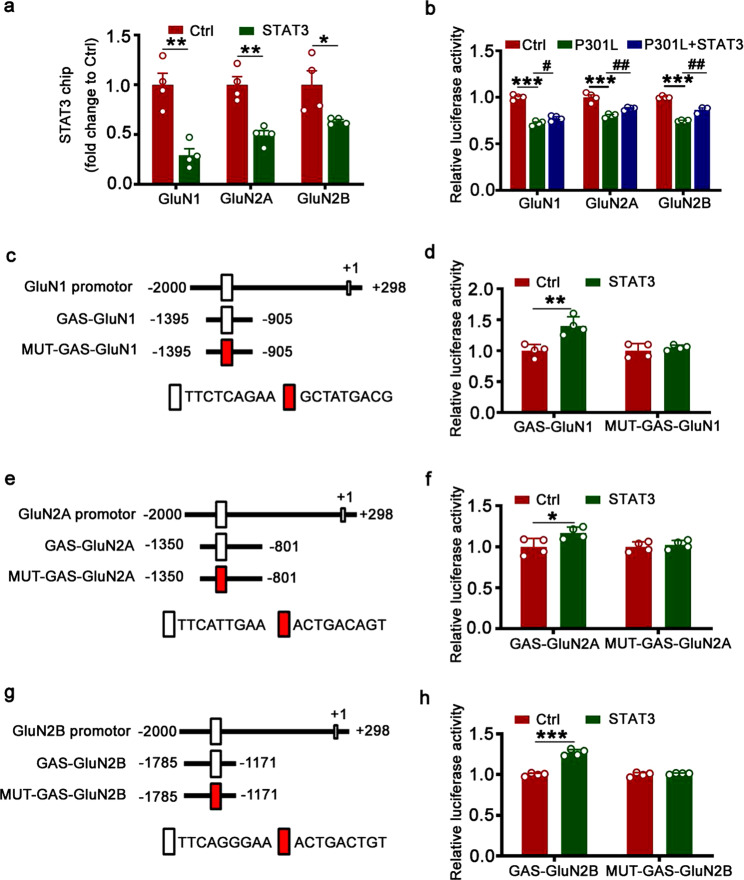
STAT3 binds to NMDAR promoters and promotes the expression of NMDARs. **a** Overexpression of AAV-P301L decreased binding of STAT3 to the promoter regions of GluN1, GluN2A, and GluN2B genes in hippocampal CA3 extracts, as measured by chromatin immunoprecipitation assay (ChIP). Two-tailed Student’s *t* test, [GluN1] *p* = 0.002, [GluN2A] *p* = 0.002, [GluN2B] *p* = 0.041, *n* = 4. **b** Overexpression of wild-type STAT3 (WT-STAT3) ameliorated P301L-induced reduction of the transcription activity of NMDARs in HEK293 cells. Two-way analysis of variance (ANOVA) followed by the Bonferroni’ s post hoc test, [GluN1] ***p < 0.001, #*p* = 0.049; [GluN2A] ***p < 0.001, ##*p* = 0.006; [GluN2B] ***p < 0.001, ##*p* = 0.002; *n* = 4. **c**–**h** Diagrams show the predicted GAS promoter element (GASs) for STAT3 in the promoter (−2000 ± 298 bp) of GluN1 (**c**), GluN2B (**e**), and GluN2A (**g**). The GAS or the mutant (MUT) plasmids were co-transfected respectively with WT-STAT3 or its empty vector (Ctrl) into HEK293 cells for 24 h, and then the luciferase activity was measured (right panels, **d**, **f**, **h**). Two-tailed Student’s *t* test; **d** *p* = 0.004; **f** *p* = 0.035; **h** *p* < 0.001; *n* = 4. Data were presented as mean ± SEM

We measured the level of synapse-related protein to explore the molecular mechanisms underlying STAT3 ameliorating P301L-hTau-induced dendritic plasticity impairments. P301L-hTau accumulation or STAT3 knockdown by AAV-Cre in mice decreased the protein and mRNA levels of postsynaptic proteins NMDARs type 1 (GluN1), GluN2A, and GluN2B (Supplementary Fig. S5), while overexpression of STAT3 by AAV-STAT3 substantially restored the reduction of protein and mRNA levels of the NMDARs induced by P301L-hTau (Supplementary Fig. S5d–f). Further studies by chromatin immunoprecipitation (CHIP) assay demonstrated that overexpression of P301L in hippocampus markedly decreased binding of STAT3 to the promoter of GluN1, GluN2A, or GluN2B (Fig. 6a), and upregulating STAT3 ameliorated the reduced transcriptional activity of the NMDARs induced by P301L (Fig. 6b). These data demonstrated that STAT3 activation promoted NMDAR expression by directly binding to the promoters.

To explore how STAT3 promotes the expression of NMDARs, we screened potential binding sites of STAT3 in the promoter regions of GluN1, GluN2A, and GluN2B in a transcription factor database. We found one conserved GAS promoter element for STAT3 binding in the promoter regions of GluN1, GluN2A, or GluN2B, respectively. To prove whether the GAS promoter element of GluN1, GluN2A, or GluN2B genes is specific for STAT3, we constructed luciferase reporters containing GAS elements on the NMDAR promoters (Fig. 6c, e, g). After co-transfection of specific GAS element reporters with STAT3 into HEK293 cells, we found that co-expression of STAT3 with GAS on NMDARs increased luciferase activity (Fig. 6d, f, h). Furthermore, expression of mutant GAS on NMDARs abolished STAT3-induced activation of luciferase activity (Fig. 6d, f, h). These data suggest that STAT3 promotes NMDARs expression by binding to the specific GAS element.

### Inhibition of STAT1 acetylation does not reverse the learning and memory impairments induced by P301L-hTau

Our present data showed that acetylated STAT1 detained STAT3 in the cytoplasm and thus inhibited STAT3 activity (Figs. 2 and 3). To investigate the role of STAT1 acetylation in regulating expression of NMDARs and cognitive ability, we constructed non-acetylation STAT1 mutant (K410/413R-STAT1) AAV (AAV-STAT1KR) virus and co-infused the virus with AAV-P301L-hTau into the hippocampal CA3 of 2-month-old C57 mice for 1 month. Unexpectedly, we found that, co-expression of KR-STAT1 improved spatial learning ability (Supplementary Fig. S6), however, did not attenuate P301L-hTau-induced spatial memory deficits though spatial memory shown an improving trend (Supplementary Fig. S6d–j), and LTP suppression and spine density reduction had no change compared with P301L (Supplementary Fig. S6k–n). GluN1 and GluN2A protein and mRNA levels also showed no significant change relative to those of P301L-hTau group (Supplementary Fig. S7).

Our previous study showed that tau accumulation activated STAT1 to inhibit NMDARs transcription.^26^ STAT1 acetylation inhibited STAT1 entry into the nucleus.^42^ Here, transfection with K410/413R-STAT1 (STAT1KR) may not only increase STAT3 protein level in the nuclear fraction, but also promote STAT1 entry into the nucleus. To prove this hypothesis, P301L-hTau and STAT1KR plasmids were co-transferred in HEK293 cells, and whole-cell lysis and nuclear fraction were extracted. Western blotting results showed that overexpression of STAT1KR increased both exogenous and endogenous protein levels of STAT1 and its phosphorylation at Y701 (STAT1 activated form) in the nuclear fraction significantly (Supplementary Fig. S8a, b). The luciferase assay also showed that STAT1KR increased STAT1 transcriptional activity (Supplementary Fig. S8c). Co-overexpression STAT1 and WT-STAT3 had no effect in the luciferase activity of NMDARs (Supplementary Fig. S8d). These data suggested that the inhibition of STAT1 acetylation resulted in activating STAT1, which antagonized the promoting effects of STAT3 on the transcription of NMDARs.

### JAK2 kinase mediates phosphorylation of STAT3 at Y705

Our previous paper reported that hTau accumulation activated tyrosine kinase JAK2, JNK, and ERK, which are reported to mediate phosphorylation of the STAT family.^26^ Here, we also found that P301L-hTau activated JAK2, JNK, and ERK (Supplementary Fig. S9a, b). Simultaneous inhibition of JAK2 by JAK2 inhibitor TG-101348 (JAK2I) or JAK2 siRNA but not JNK or ERK inhibitor (SP600125 or FR180204) abolished P301L-hTau-induced STAT3 phosphorylation at pY705 in the cell extracts (Supplementary Fig. S9c–f).

## Discussion

While the amount of abnormal tau protein aggregation into neurofibrillary tangles is positively correlated with the degree of neurodegeneration and memory impairment in tauopathies,^5,43,44^ how tau accumulation affects synaptic proteins has been unclear until now. By overexpression of P301L-hTau (P301L), the human tau with the most common FTDP-17 mutation,^45^ we found that P301L accumulation caused STAT3 retention in the cytoplasm by acetylating STAT1 and increasing the binding with STAT3 in the cytoplasmic fraction, which inhibited the translocation of STAT3 into the nucleus and inactivated STAT3. Knockdown of STAT3 in the STAT3^flox/flox^ mice by AAV-Cre mimicked overexpressing P301L-induced synaptic and cognitive deficits via inhibition of the expression of GluN1, GluN2A, and GluN2B by binding to their specific promoter elements. We also demonstrated that overexpressing STAT3 attenuated P301L-induced synaptic and cognitive deficits. These findings reveal that intracellular accumulation of P301L-hTau causes memory deterioration through suppression of NMDARs expression via inactivation of STAT3, which discloses a novel mechanism for tau-related synapse and memory impairments.

The STAT protein family includes seven members: STAT1-4, STAT5a, STAT5b, and STAT6, which serve as transcription factors that bind to the target gene’s DNA promoter region.^27–29^ STAT1 and STAT3 are linked to an increased pro-inflammatory response. STAT1 is largely restricted to mediating the effects of IFNs, animals that lack STAT1 are exquisitely sensitive to microbial infections; while STAT3 mediates the effects of IL-6 and other gp130 ligands, and regulates the host response to bacterial infection. STAT3 loss could result in embryonic lethality. In addition, STAT1 and STAT3 have different roles in cell apoptosis and the progression of malignant tumors. STAT1 plays an important role in growth arrest and promoting apoptosis, and is implicated as a tumor suppressor; while STAT3 suppresses apoptosis and promotes tumor cell proliferation, and plays a critical role in the invasion and metastasis of malignant tumors. It may also be involved in the malignant transformation of cells. In addition to its established role as an oncogene in cancer, STAT3 regulates mitochondrion functions and gene expression through epigenetic mechanisms. Our recent paper showed that overexpressing hTau upregulated STAT1 activity and inhibited the transcription of NMDARs.^26^ As STAT1 and STAT3 have a similar DNA-binding domain GAS element,^28,29^ here we found both in vivo and in vitro that overexpressing P301L induced STAT3 phosphorylation at tyrosine 705. The increased phosphorylated level is reported to lead STAT3 to transport from the cytoplasm into the nucleus and initiate transcription of the corresponding target gene.^36,37^ However, STAT3 in the nuclear fraction decreased, which suggested that STAT3 transport into the nucleus was inhibited by overexpressing P301L. By using multiple measures, including phosphorylation (pS727-STAT3), which regulates the transcriptional activation of STAT3,^46,47^ dimerization, EMSA, and luciferase activity assay, we provide strong evidence to show that P301L accumulation inactivates STAT3.

Phosphorylated STAT3 at Tyr705 induces not only STAT3 to form a homologous dimer but also a heterodimer with STAT1.^48,49^ First, we found that the interaction between STAT1 with STAT3 increased in the cytoplasm but decreased in the nucleus. It is reported that the acetylation of STAT1 increased the binding with NF-κB p65 and inhibited its incorporation into the nucleus.^38^ By mass spectrometry, two STAT1 acetylated sites K410 and K413 were identified, and overexpressing the pseudoacetylated STAT1 mutant at both sites (K410/413Q-STAT1) decreased STAT3 in the nuclear fraction, while overexpressing the non-acetylated STAT1 mutant at both sites (K410/413R-STAT1) attenuated the reduction of STAT3 in the nuclear fraction induced by P301L. We also found that overexpressing K410/413Q-STAT1 decreased STAT3 transcriptional activity, while overexpressing K410/413R-STAT1 ameliorated the decreased P301L-induced STAT3 activity. These data suggest that P301L overexpression acetylated STAT1 and STAT1 acetylation bound with STAT3 in the cytoplasm and thus caused STAT3 retention in the cytoplasm, finally resulted in inactivation of STAT3. We also noticed that only overexpressing K410/413R-STAT1 did not change the subcellular localization of STAT3, but it might be that the acetylated STAT1 level was at a low basal level, a subject that needs further study.

To identify the exact binding element(s) of STAT3 on NMDAR promoters, we constructed GAS promoter elements in NMDAR promoter regions for the luciferase activity assay. As the specific sequence of the GAS element for STAT1 is TTC(N2-4)GAA, and for STAT3 is TTC(N3)GAA,^28,29^ we observed only one conserved GAS promoter element for STAT3 binding in the promoter regions of GluN1, GluN2B, or GluN2A, though there are two conserved GAS promoter elements for STAT1 binding in the promoter regions of GluN1 and GluN2B, and four GAS promoter elements in GluN2A.^26^ By transfection with GAS-NMDARs or MUT-GAS-NMDARs (mutant) plasmid construct, we found that STAT3 positively regulates the luciferase activity of NMDRAs. Combined with data from Chip, PCR, and Western blotting, we concluded that STAT3 positively regulates the expression of NMDARs. Though STAT1 and STAT3 both belong to the same protein family and employ similar GAS elements to regulate downstream gene expression as a transcriptional factor, we first found that STAT3 and STAT1 nevertheless have opposite regulatory roles in NMDARs expression: STAT3 positively regulates NMDARs expression, while STAT1 negatively regulates NMDARs expression.^26^

Besides the presence of abnormal tau protein, some tau-dominated polyprotein proteinopathies contain other abnormal protein accumulations, including TDP-43 and α-synuclein. We found that neither TDP-43 nor α-synuclein affected the activity of STAT3, and they also did not change the expression of NMDARs. These negative results suggest that the inactivation of STAT3 is specific for Tau accumulation.

To investigate the mechanisms by which overexpression of P301L induced an increase of STAT1 acetylation, we detected the proteins CBP, p300, or HDAC2, which have acetylase or deacetylase activity.^38–40^ However, only CBP protein level was significantly decreased in P301L overexpression, which suggests that CBP, p300, or HDAC2 are not involved in STAT1 acetylation. Tau protein itself has been reported to possess acetyltransferase activity, such that the repeat domain K18 mediates the acetyltransferase action.^41^ We found that P301L interacted with STAT1 and thus increased the STAT1 acetylation level, but overexpressing K18(−) (K18-deleted P301L mutant) had no effects on the acetylated level of STAT1. These in vitro and in vivo data suggested that P301L accumulation acetylated STAT1. We also found that here, similar to our earlier demonstration that hTau accumulation activated JAK2,^26^ that P301L also activated JAK2 to phosphorylate STAT3. This implies that activation of JAK2/STAT signaling pathway is a common effect of Tau accumulation.

As STAT1 acetylation played a key role in the nuclear localization and transcriptional activity of STAT3, we infected AAV-STAT1KR (410/413R-STAT1) with AAV-P301L virus into C57 mice and investigated whether non-acetylated STAT1 overexpression attenuated P301L-induced synaptic and cognitive deficits. Unexpectedly, overexpression of STAT1KR had no effect on the P301L-induced impairment of dendritic plasticity and memory ability. This was the result of inhibition of STAT1 acetylation promoted STAT3 transport into the nucleus and activation of STAT3, while STAT1 in the nuclear fraction and its activity was also increased by non-acetylated STAT1. We recently reported that STAT1 negatively regulates the expression of NMDARs.^26^ The negative effects of STAT1 activation antagonized the positive effects of STAT3 activation in NMDARs expression, so NMDARs expression (mRNA and protein level) showed no change and memory also did not been improved. The reason for non-acetylated STAT1 favor STAT1 nuclear translocation is that, as a positive charge is required for STAT1 to associate with nuclear translocation, Lys410 and lys413 are embedded within the nuclear localization sequence of STAT1, such that acetylation of these lysines would neutralize their positive charge and preclude STAT1 nuclear accumulation.^42,50^

By combining the present data with those in our recent study,^26^ we propose the following “trade-off hypothesis” as the molecular mechanism by which the accumulation of tau induces synaptic deficits. We propose that tau accumulation activates the JAK2/STAT1 signaling pathway to phosphorylate STAT1 at Y701 (Supplementary Fig. S10), promotes STAT1 to form a homodimer for transport into the nucleus, and thus activates STAT1 to suppress the expression of NMDARs.^26^ To rectify the adverse consequences of STAT1 activation, tau acetylates STAT1 to inhibit STAT1 nuclear translocation. However, acetylated STAT1 promotes STAT1 binding with STAT3 to form a heterodimer in the cytoplasm, which prevents STAT3 transport into the nucleus even though JAK2 phosphorylates STAT3 at Y705. Tau accumulation inhibits NMDARs expression via upregulation of STAT1 activity and downregulation of STAT3 activity, ultimately leading to synaptic dysfunction and cognitive deficits (Supplementary Fig. S11). We also noticed that STAT3 overexpression reduced the insoluble tau, which also contributed to cognitive improvements in addition to increased NDMAR protein levels. In a word, our study suggests that STAT3 will hopefully serve as a potential pharmacological target for tauopathies treatment.

In the present study, we found that overexpressing P301L-hTau induced synaptic and cognitive deficits by decreasing NMDARs expression. However, whether the mechanisms found here is due to mutation of tau at P301L, or mere overexpression of htau in mouse brain, or due to hyperphosphrylation of tau produced from the overexpression of the mutated protein, is not clear, which is waiting for further study. It may be interesting in future studies to explore whether other different kind of tau protein or tau phosphorylation (sites or level) is involved in this synaptic mechanism. In addition, by stereotaxic infusion of AAV-P301L-hTau, we could only achieve 2.5-times increase of tau proteins in the hippocampal CA3 region, which was closer to the observed increased level in the AD brains, and much lower than that (~13-fold) in Tg4510 transgenic mice.

## Methods and materials

### Antibodies and reagents

The antibodies used in the present study are listed in the Supplementary Table S1. TG-101348 (special JAK2 inhibitor, from MCE), JAK2 siRNA (sc-39099, from Santa Cruz Biotechnology), SP600125 (the inhibitor of JNK1, from Santa Cruz), and FR180204 (the inhibitor of ERK1, from Santa Cruz) were purchased. PcDNA3.0 vector-coded wild-type STAT1 (WT-STAT1) plasmid was the gift of Dr Xiao-Yuan Li (Institute of Biomedical Sciences, Academia Sinica, Taiwan), and PcDNA3.0 vector-coded wild-type STAT3 (WT-STAT3) plasmid was purchased from Shangdong Vigene Bioscience Biotechnology, co., LTD. EGFP-N1 vector coded mutant full-length human tau (P301L-hTau) plasmid, WT-STAT1 plasmid was mutated to pseudoacetylated STAT1 plasmids (single or double lysine sites (410, 413) mutated to glutamine, K410Q-, K413Q-, K410/413Q-STAT1) or unacetylated STAT1 plasmids (single or double lysine site (410, 413) mutated to arginine, K410R-, K413R-, K410/413R-STAT1) coded into RFP-N1 vector by Shanghai Baicheng Biotechnology, co., LTD.

### Animals

Male C57 mice were purchased from the Animal Center of Tongji Medical College, Huazhong University of Science and Technology. STAT3^flox/flox^ mice (B6; 129S-STAT3tm1Mam/Mmjax) and Tg4510 mice (FVB-Fgf14Tg(tetO-MAPT*P301L)4510Kha/JlwsJ) were purchased from Jackson Laboratory. All mice were kept at 22 ± 2 °C on 12 h light–dark cycles with ad libitum access to food and water. All animal experiments were performed according to the “Policies on the Use of Animals and Humans in Neuroscience Research” revised and approved by the Society for Neuroscience (USA) in 1995, and the Guidelines for the Care and Use of Laboratory Animals of the Ministry of Science and Technology of the People’s Republic of China. The Institutional Animal Care and Use Committee at Tongji Medical College, Huazhong University of Science and Technology approved the study protocol.

### Stereotaxic brain injection

Adeno-associated virus coded for human mutant full-length P301L-hTau (AAV-P301L) with the N-terminal fused with enhanced green fluorescent protein (eGFP), and the control AAV-eGFP, AAV-STAT3, AAV-K410/3R-STAT1 virus were purchased from OBio Biologic Technology Co., Ltd. AAV-Cre was purchased from Shanghai Genechem Co., Ltd. The titer of AAV-P301L was 1.3 × 10^13^ v.g./ml, that of AAV-STAT3 virus was 1.2 × 10^13^ v.g./ml, and the titer for AAV-K410/3R-STAT1 virus was 1.1 × 10^13^ v.g./ml. The titer of AAV-Cre or the control virus was 7.9 × 10^12^ v.g./ml. All AAV viruses were driven by CMV promoter. The in vivo overexpression efficiency was measured by immunohistochemical staining and Western blotting 1 month later after injection of the virus into the hippocampal CA3 region of mice. After positioned in a stereotaxic instrument, 2-month-old C57 or STAT3^flox/flox^ mice were bilaterally injected the virus into the hippocampal CA3 region (AP ± 2.0, ML −1.5, DV −2.0) at a rate of 0.10 μl/min. The needle syringe was left in place for ~3 min before being withdrawn. The injection did not significantly change the normal activity or increase the death rate of the mice compared with the non-injected controls.

### Behavioral tests

Approximately one month after brain infusion of the virus, the Morris water maze (MWM) test was used to assess spatial learning and memory. For spatial learning, mice were trained to find a hidden platform in the water maze for 6 consecutive days, four trials per day with a 30-s interval from 14:00 to 20:00 p.m. On each trial, an operator let the animal face the wall of the pool and started from one of the four quadrants. When the animal climbed on the platform, the trial ended. If the mice did not locate the platform within 60 s, operators guided them to the platform and stayed for 30 s. The spatial memory was tested 24 h after the last training. The longer a mouse stayed in the quadrant where the platform previously located, the higher the score for spatial memory. The swimming path and the latency to find the platform or times passing through the previous platform-located quadrant (during test phase) were recorded by a video camera. The camera was fixed to the ceiling, 1.5 m from the water surface, and connected to a digital-tracking device, which attached to an IBM computer.

The contextual fear conditioning test was performed according to our previously established procedure. Briefly, prior to experimentation, in order to adapt to the environment, the mouse was kept in the cage for 3 min. The animal then received training via 3-min unsignaled foot-shocks (one shock at the 1st min, three shocks at the 2nd min, and eight shocks at the 3rd min; 0.5 mA, 2-s duration, and 1 min apart). LTM was tested, respectively, 24 h post-training by placing the animal back into the conditioning chamber for 3 min and measuring the freezing time.

The new object recognition test was performed as following: a 5-min habituation without objects was conducted. After 1 h, an object familiarization phase with two of Object A for 5 min was conducted. A further 5-min testing period with one of Object A and Object B, which were randomly assigned as A and B, was conducted after 1 h later. A blinded investigator scored object recognition times. The novel object recognition ratio was calculated as: [(time novel object)/(time novel object + time familiar object)]. Object exploration was defined as active investigation of an object within ~2 cm or less of its nose.

### Preparation of nuclear fractionation

According to instructions of the manufacturer, the nuclear extracts were prepared using the nuclear extraction kit (Signosis, Inc., Sunnyvale, CA, USA). Briefly, Buffer I working reagent was added to the culture dish and rocked at 200 rpm on a shaking platform at 4 °C for 10 min. The HEK293 cells were collected and centrifuged at 12,000 rpm at 4 °C for 5 min. The supernatant was discarded, and the pellets were re-suspended by adding Buffer II working reagent. For tissues, the hippocampal CA3 areas (location of viral infected) were rapidly cut into small pieces, added Buffer I working reagent, and homogenized at 4 °C until a single cell suspension was observed microscopically. After spinning at 500 g at 4 °C for 5 min, and the supernatant was removed, the cell pellets were re-suspended in Buffer I working reagent, and the preparation rocked at 200 rpm on a shaking platform at 4 °C for 10 min. The cells were then centrifuged at 10,000 rpm at 4 °C for 5 min, and the pellets were re-suspended by adding Buffer II working reagents. Lastly, the cell lysate was shaken at 200 rpm on a platform at 4 °C for 2 h. After centrifugation at 12,000 rpm at 4 °C for 5 min, the supernatant (nuclear extract) was collected and stored at −80 °C until use.

### Electrophoresis mobility shift assay (EMSA)

The nonradioactive EMSA-STAT3 Kit was purchased from Signosis (Sunnyvale, CA, USA). EMSA was performed according the instruction of the manufacturer. Briefly, a biotinized oligonucleotide probe, containing a STAT3 binding site, was incubated with the samples, and then, the samples were separated on a non-denaturing polyacrylamide gel and transferred to nylon membranes. UV cross-linking was used to immobilize the transferred oligonucleotides. In order to detect the oligonucleotides, Streptavidin-HRP was added to the membrane, and the blots were developed by ECL according to the instructions of manufacturers. Excess amounts of unlabeled cold probe, containing STAT3 binding site, was used to perform a competition experiment.

### ChIP assay

The DNA and protein were cross-linked with formaldehyde (1%) for 10 min, and washed three times and scraped into cold PBS with protease inhibitors. After centrifugation, the cell pellet was re-suspended in buffer (in mM): 20 HEPES, pH 7.9, 420 NaCl, 1.5 MgCl_2_, and 0.2 EDTA, with 25% glycerol and protease inhibitors, incubated for 20 min on ice, and centrifuged. The pellet (nucleus) was re-suspended in breaking buffer (in mM): 50 Tris-HCl, pH 8.0, 1 EDTA, 150 NaCl with 1% SDS, 2% Triton X-100, and protease inhibitors, and sonicated for 5–10 s, and Triton buffer (in mM): 50 Tris-HCl, pH 8.0, 1 EDTA, 150 NaCl, with 0.1% Triton X-100 was added. An aliquot was reserved as the input, and the remainder was divided to immunoprecipitate with control mouse IgG (Millipore,) or STAT3 (Abcam) antibody and incubated with protein G beads. After washed three times in Triton buffer, the samples were added with SDS buffer consisted of 62.5 mM Tris-HCl, pH 6.8, 200 mM NaCl, 2% SDS, 10 mM DTT, 2 μl of proteinase K (40 mg/ml), and then, samples were vortexed. To reverse cross-linking, samples were incubated at 65 °C overnight. After isolated using phenol/chloroform extraction, DNA was re-suspended in distilled H_2_O. Primers used for ChIP PCR were as following: GluN1 forward and reverse primer, 5′-TAGCATTGGCATTGACCC-3′, 5′-GCTGGTGCGGTGATGTGA-3′; GluN2A forward and reverse primer, 5′-TCGGCTTGGACTGATACGTG-3′, 5′-AGGATAGACTGCCCCTGCAC-3′; GluN2B forward and reverse primer, 5′-TCTCCACCGTGCTGATGT-3′, 5′-CTCTCCGAGTCTACCTGTTC-3′. PCR products were analyzed by 2% agarose gel electrophoresis.

### Statistical analysis

All data were collected and analyzed in a blinded manner. Data were expressed as mean ± SEM. Statistical analysis was performed using Student’s *t*-test (two-group comparison), one-way ANOVA or two-way repeated measures ANOVA followed by the Bonferroni’s post hoc test with SPSS 12.0 statistical software (SPSS Inc. Chicago, IL, USA). A *p* value of <0.05 was considered as statistical significance in all experiments.

## Supplementary information

supplementary materials

## Data Availability

The data sets used for the current study are available from the corresponding author upon reasonable request.
