# Supplementary material for: STAT3 ameliorates cognitive deficits by positively regulating the expression of NMDARs in a mouse model of FTDP-17
Source: Signal Transduct Target Ther. 2020 Dec 26;5:295. doi: 10.1038/s41392-020-00290-9 (PMC7762755; doi:10.1038/s41392-020-00290-9)
Supplement: Supplementary file 1 — supplementary materials [file 41392_2020_290_MOESM1_ESM.pdf]

Supplementary Materials for

STAT3 ameliorates cognitive deficits by positively regulating the expression of NMDARs in a mouse model of FTDP-17

Xiao-Yue Hong[1‡], Hua-Li Wan[1‡], Ting Li[1], Bing-Ge Zhang[1], Xiao-Guang Li[2], Xin Wang[1], Xiao Li[1], Qian Liu[1], Chong-Yang Chen[1], Ying Yang[1], Qun Wang[1], Shu-Peng Li[3], Hao Yu[4], Jian-Zhi Wang[1], Xi-Fei Yang[5]*, Gong-Ping Liu[1,6]*

Correspondence to: Xi-Fei Yang (xifeiyang@gmail.com) or Gong-Ping Liu (liugp111@mail.hust.edu.cn)

This PDF File includes:

Materials and Methods

Figures. S1 to S11

Tables S1and S2

Materials and Methods

Electrophysiological analysis

A Leica VT1000S vibratome (Milton Keynes, UK) was used to cut horizontal brain slices (400 μm) containing the dorsal hippocampus at 4 ºC in artificial cerebrospinal fluid (aCSF), which consisted of: 126 mM NaCl, 3 mM KCl, 1.25 mM NaH_2PO_4, 24 mM $NaHCO_3$, 2 mM $MgSO_4$, 2 mM $CaCl_2$ and 10 mM glucose (pH 7.4; 305 mOsm), and saturated with carbogen (95% O_2 and 5% CO_2). Immediately after slicing, sections were transferred and maintained in an interface chamber continuously perfused with aCSF, and the slices were equilibrated at least for 30 min prior to recording at room temperature.

For extracellular recordings, slices were placed in the interface recording chamber at 32 ºC and the perfusion rate was normally 3 ml/min, while maintaining a thin film of aCSF covering the slice to make sure applied substances could diffuse into the area recorded. Field potentials were amplified with Neurolog AC-coupled NL 104 preamplifiers (Digitimer Ltd, Welwyn, UK). The excitatory postsynaptic potential (fEPSP) was recorded by a 0.1-MΩ tungsten monopolar electrode from the dendritic layer of the stratum radiatum of the CA3 field following electrical stimulation of the mossy-fiber pathway. The electrical pulses were delivered using a bipolar platinum/iridium electrode (25 μm wire-diameter, at an inter-wire distance of 100 μm, World Precision Instruments, USA). The fEPSP was quantified by 30 % of the maximum slope of its rising phase. We used theta-burst stimulation (TBS), which consisted of 4 pulses at 100 Hz, repeated 3 times with a 200-ms interval, to induct long-term potentiation (LTP). LTP magnitude was expressed as the mean percentage of baseline fEPSP initial slope.[1,2]

Cell culture

HEK293 (human embryonic kidney293) were cultured in Dulbecco's Modified Eagle's medium (DMEM), supplemented with fetal bovine serum (10%, v/v) and penicillin/streptomycin (1%), in a humidified atmosphere containing 5% CO_2 at 37 ºC. The cells grew in plates for 24 h, and then the indicated plasmid(s) were transfected into the cells using Lipofectamine2000 according to the instructions of manufacturers.

For primary neuron cultures, 18-day embryonic (E18) hippocampus of rat was seeded on 6-well plates at 30,000-40,000 cells per well, which coated with Poly-D-Lysine/Laminin (Bioscience) in neurobasal medium (Invitrogen) supplemented with B27 (2%)/ glutamine (0.5 mM) / glutamate (25 mM). Half the culture medium was changed every 2 days with neurobasal medium supplemented with B27 (2%) and glutamine (0.5 mM). All cultures were kept at 37 ºC in a humidified 5% CO_2-containing atmosphere. After cultured for 7 to 17 div, more than 90% of the cells were neurons, which was verified by positive staining for the neuronal specific marker MAP2 (dendritic marker, Millipore).

Preparation of insoluble tau

Insoluble tau aggregates were isolated from the virus infected-hippocampal tissue by a modification of a published procedure. Brain tissues were homogenized in lysis buffer (in mM): Tris-HCl 10, NaCl 150, NaF 20, Na_3VO_4 1, EGTA 2, Triton X-100 0.5%, and SDS 0.1% with protease inhibitor mixture and centrifuged at 13,000 × g for 20 min. The resulting supernatant was designated as the soluble tau fraction. The pellet was resuspended in 1% SDS buffer with 10 times ultrasonication and designated as insoluble aggregated tau.

Western blotting

10% sodium dodecyl sulfate-polyacrylamide gel electrophoresis (SDS-PAGE) was used to separate equal amounts of protein, and the separated proteins were transferred onto nitrocellulose membranes. For analysis of STAT3 dimerization, cell lysates were incubated with 1 mM DSS, blocked with 0.5 mM NH_4OH for 20 min, and then, used for Western blotting.[3] The membranes were blocked in non-fat milk (5%) at 20 ºC for 1 h and then incubated with primary antibody (Supplementary Table S1) at 4 ºC overnight. Blots were then incubated with IRDye 800CW-conjugated affinity-purified anti-mouse IgG (Rockland) or IRDye 800CW anti-rabbit IgG secondary antibody (Rockland) at 20 ºC for 1 h. Odyssey Infrared Imaging System (Licor Biosciences, Lincoln, NE, USA) was used to visualize immunoreactive bands.

Reverse transcription and real-time quantitative PCR

According to manufacturer's instruction (TaKaRa, Dalian, China), reverse transcription and real-time quantitative PCR were carried out. The PCR system contained $MgCl_2$ (3 mM), forward and reverse primers (0.5 μM), SYBR Green PCR master mixes (2 μl) and cDNA (2 μl), and the standards for each gene. A Rotor Gene 300 Real-time Cycler (Corbett Research, Sydney, Australia) was used to assay the samples. Glyceraldehyde-3-phosphate dehydrogenase (GAPDH), the housekeeping gene which was not changed by the treatments, was used to normalize the expression level of the interest gene. PCR primers employed in the present study are as follow: Mmu-GluA1 forward and reverse primers, 5'-CAATGACCGCTATGAGGG-3' and 5'-AAGGACTGAAACGGCTGA-3'; mmu-GluA2 forward and reverse primers, 5'-GTGTCGCCCATCGAAAGTG-3' and 5'-AGTAGGCATACTTCCCTTTGGAT-3'; mmu-synapsin1 (Syn1) forward and reverse primers, 5'- AGGACGAGGTGAAAGC-3' and 5'-TCAGTCGGAGAAGAGG-3'; mmu-synaptotagmin1 (Syt1) forward and reverse primers, 5'- CCATAGCCATAGTTGC-3' and 5'-GTTTCAGCATCGTCAT-3'; mmu-GluN1 forward and reverse primers, 5'- GTCCACCAGACTAAAGA-3' and 5'-TCCCATCATTCCGT-3'; mmu-GluN2A forward and reverse primers, 5'-CTTTTGAGGACGCC-3' and 5'- AAATGAGACCCGATG-3'; mmu-GluN2B forward and reverse primers, 5'- GGCTGACTGGCTACG-3' and 5'- CTTGGGCTCAGGGAT-3'; mmu-GAPDH forward primer 5'-GGAGCGAGATCCCTCCAAAAT-3' and reverse primer 5'-GGCTGTTGTCATACTTCTCATGG-3'.

Luciferase reporter assay

Activity of the transcription factors (TFs) was analyzed with the specific luciferase reporter vector pSTAT3-Luc (Signosis). This vector contains a cis-element (DNA binding sequence), a minimal promoter, and a firefly luciferase gene. The activated transcription factors binds to the cis-element and transactivates expression of the luciferase gene correlating with the measured luciferase enzyme activity. Therefore, the luciferase activity in this assay represents activation of the transcription factor. Briefly, HEK293 cells were transfected with P301L-hTau plasmid or its empty vector control in combination with pSTAT3-Luc reporter construct and pRL-TK for 48 h. Then the cells were washed and lysed in 100 μl of the 1×CCLR (Promega). Luciferase activity was measured according to the manufacturer's instruction (Promega). The activity of TF (i.e.

firefly luciferase) was normalized to transfection efficiency by using Renilla luciferase activity (pRL-TK).

To generate luciferase reporter plasmids of GluN1, GluN2A or GluN2B promoter, after copied from the mouse genomic DNA, PCR fragments were subcloned into pGL3 basic luciferase expression vector (Promega, Madison, WI) between the BglII and NcoI sites. The GeneTailor system (Invitrogen) was used to introduce mutation of the pGL3-GluN1/GluN2A/GluN2B luciferase plasmid. Luciferase reporter plasmids were transfected into HEK293 cells by Lipofectamine Plus (Invitrogen) according to the manufacturer's instructions. To assay the luciferase activity, pGL3-construct, P301L-hTau and pRL-TK plasmid were co-transfected into HEK293 cells, after the cells were seeded into 24-well plates one day prior to transfection. 24 h later, cells were harvested and lysed with Passive Lysis Buffer (100 μl). 20 μl cell extracts were used for luciferase activity assay by a Lumat LB9507 luminometer (Berthold) and the Dual Luciferase Reporter (DLR) assay system (Promega).

Immunohistochemistry

In brief, mice were terminally anesthetized with isoflurane and perfused through aorta with physiological saline (100 ml) followed by phosphate buffer containing 4 % paraformaldehyde (400 ml). After removed and postfixed in perfusate overnight, brains were cut into sections (30 μm) using a vibratome (Leica, Nussloch, Germany; S100, TPI), and then, sections were collected consecutively in PBS for immunohistochemistry. Free floating sections or FTDP-17 brain sections were blocked with 0.3% H_2O_2 in absolute ethanol for 30 min at room temperature, and incubated with bovine serum albumin (BSA) for another 30 min to block nonspecific sites. Primary antibodies were used to incubate with sections at 4 ºC overnight. Immunoreaction was developed and visualized with diaminobenzidine (brown color) by Histostain TM-SP kits. FTDP-17 brain sections were counterstained with hematoxylin, and mouse or human sections dehydrated through a graded ethanol series, mounted on glass slides, and sealed with glass coverslips. For each primary antibody, 3-5 consecutive sections from each brain were used. A microscope (Olympus BX60, Tokyo, Japan) was used to observe the images.

The human brain tissues used in the present study (Supplementary Table S2) were provided by Dr. K Ye of the Emory University School of Medicine, USA. The study was approved by the Biospecimen Committee. Informed consent was obtained from the subjects.

Reference

1. Fernandes, H.B. et al. Epac2 mediates cAMP-dependent potentiation of neurotransmission in the hippocampus. *J. Neurosci.* **35**, 6544-6553 (2015).

2. Wang, H. et al. Metabotropic glutamate receptors induce a form of LTP controlled by translation and Arc signaling in the hippocampus. *J. Neurosci.* **36**, 1723-1729 (2016).

3. Koshelnick, Y., Ehart, M., Hufnagl, P., Heinrich, P.C. & Binder, B.R. Urokinase receptor is associated with the components of the JAK1/STAT1 signaling pathway and leads to activation of this pathway upon receptor clustering in the human kidney epithelial tumor cell line TCL-598. *J. Biol. Chem.* **272**, 28563-28567 (1997).

Figure S1.

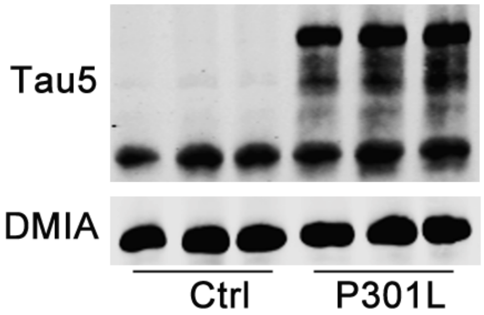

Figure S1. Total tau level increased by overexpression of P301L-hTau.
Overexpression of human tau containing the most common FTDP-17 mutation (P301L-hTau, or P301L) increased total tau (tau-5) protein level to 2.5 times, compared with the control.

Figure S2

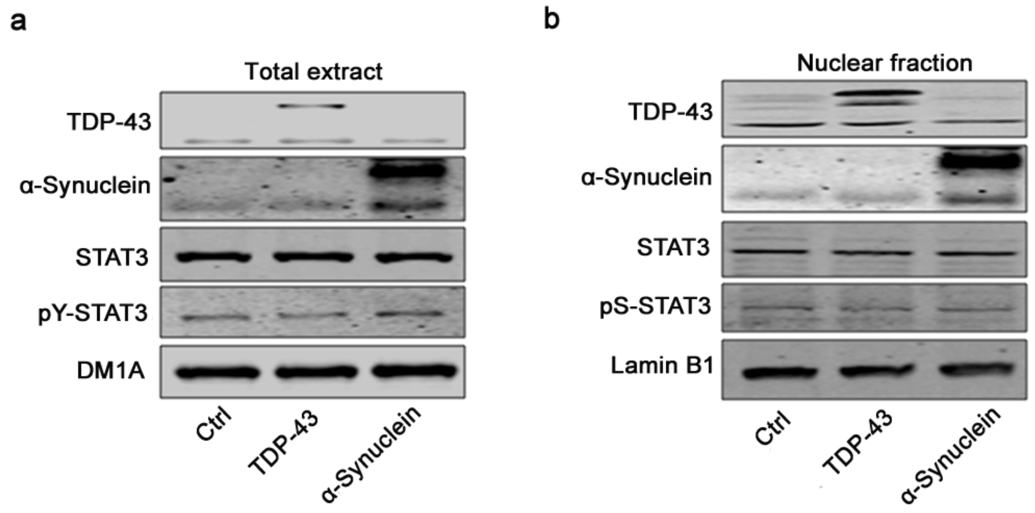

Figure S2. Inactivity of STAT3 was specific to P301L-hTau.

Overexpression of TDP43 or α-synuclein did not change the protein level of total or phosphorylated STAT3 in total extract (a) or the nuclear fraction (b).

Figure S3

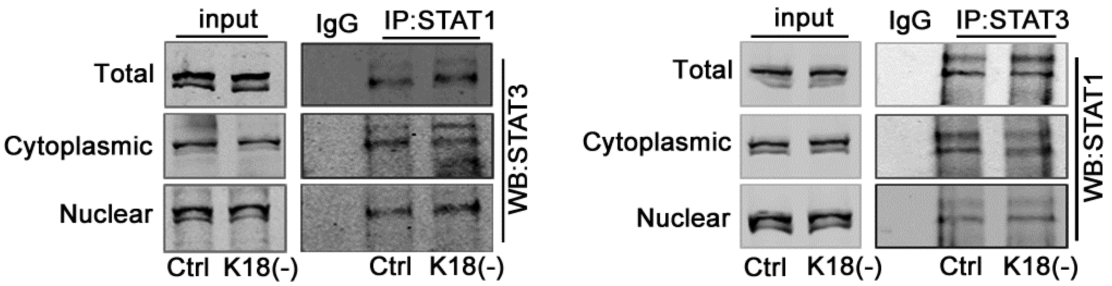

Figure S3 P301L-K18(-) had no effects in the interaction of STAT3 with STAT1 in the cytoplasmic fraction.

P301L-K18(-) (P301L tau lacking the repeats) plasmid was transfected into HEK293 cells, and the interaction of STAT3 with STAT1 in the total, cytoplasmic or nuclear fraction was detected by CO-IP**.**

Figure S4

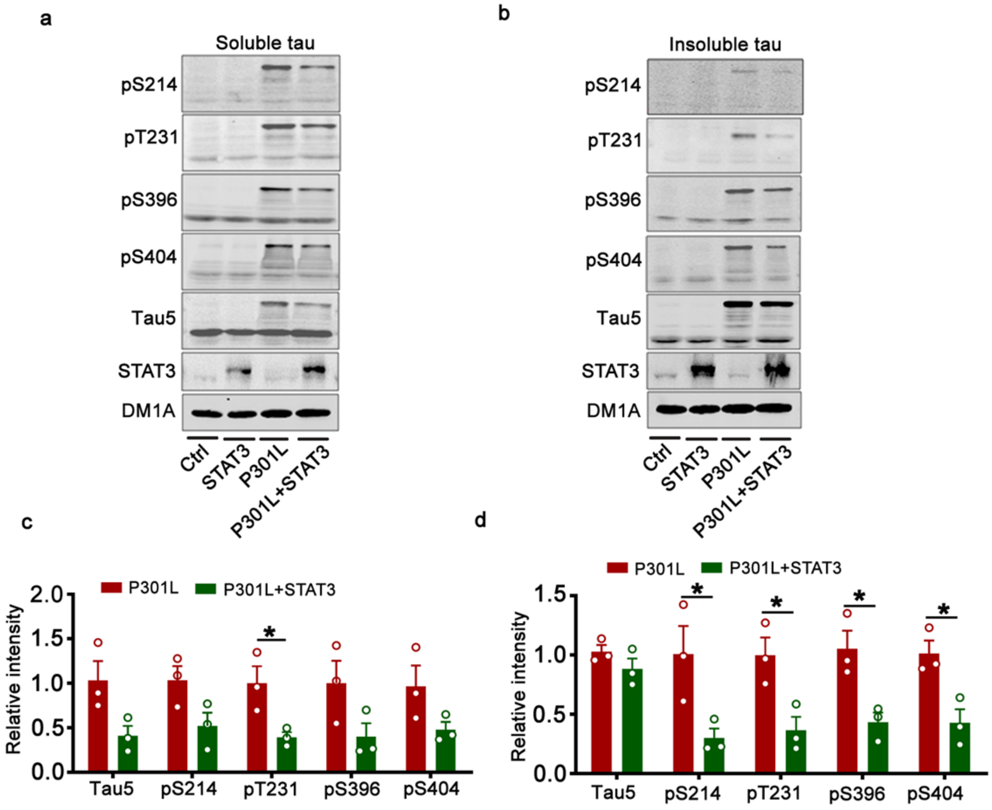

Figure S4 STAT3 decreases aggregation of insoluble tau.

AAV-P301L-hTau-eGFP (1.3×10E^{13} v.g./ml) or combined with AAV-STAT3 (1.2×10^{13} v.g./ml) were stereotaxically injected into hippocampal CA3 of 2-month-old C57 mice. After 1 month, the level of phosphorylated tau in soluble (a, c) and insoluble fraction (b, d) was detected by Western blotting. Two-tailed Student's t-test, Soluble tau [pT231] $p=0.038$; Insoluble tau [pS214] $p=0.046$, [pT231] $p=0.028$, [pS396] $p=0.023$, [pS404] $p=0.021$, $n=3$.

Data were presented as mean ± SEM.

Figure S5

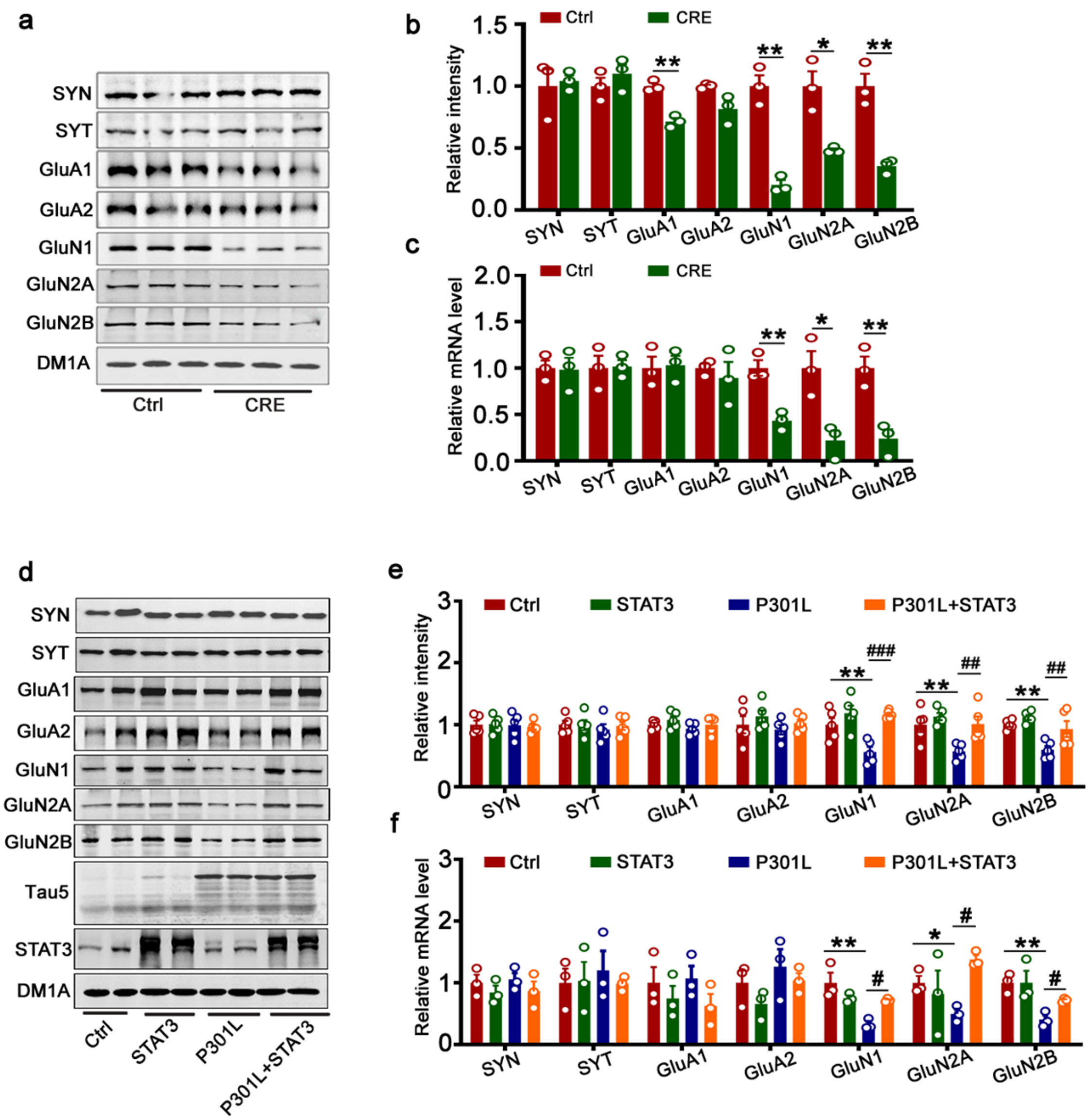

Figure S5 STAT3 positive regulates expression of NMDARs.

(a-c) AAV-Cre (5×10^{12} v.g./ml) were stereotaxically injected into the hippocampal CA3 of 2-month-old STAT3$^{flox/flox}$ mice. One month later, knockout of STAT3 decreased the protein (**a, b**) and mRNA (**c**) levels of GluN1, GluN2A and GluN2B, as detected by Western blotting or qRT-PCR. Two-tailed Student's t-test, b, [GluA1] p=0.0016;

[GluN1] p=0.0011, [GluN2A] p=0.0123, [GluN2B] p=0.0036; c, [GluN1] p=0.005, [GluN2A] p=0.021, [GluN2B] p=0.004; n=3.

(d-f) Overexpression of STAT3 ameliorated AAV-P301L-induced protein and the mRNA levels of GluN1, GluN2A and GluN2B, as detected by Western blotting or qRT-PCR in the hippocampal CA3. Two-way analysis of variance (ANOVA) followed by Bonferroni's post hoc test, e, [GluN1]** p=0.004, ### p<0.001; [GluN2A]** p=0.006, ## p=0.005; [GluN2B] **p=0.002, ##p=0.007, n=5. f, [GluN1]**p=0.001, #p=0.014; [GluN2A] *p= 0.023, #p=0.016, [GluN2B]**p=0.006, #p=0.013; n=3.

Data were presented as mean ± SEM

Figure S6

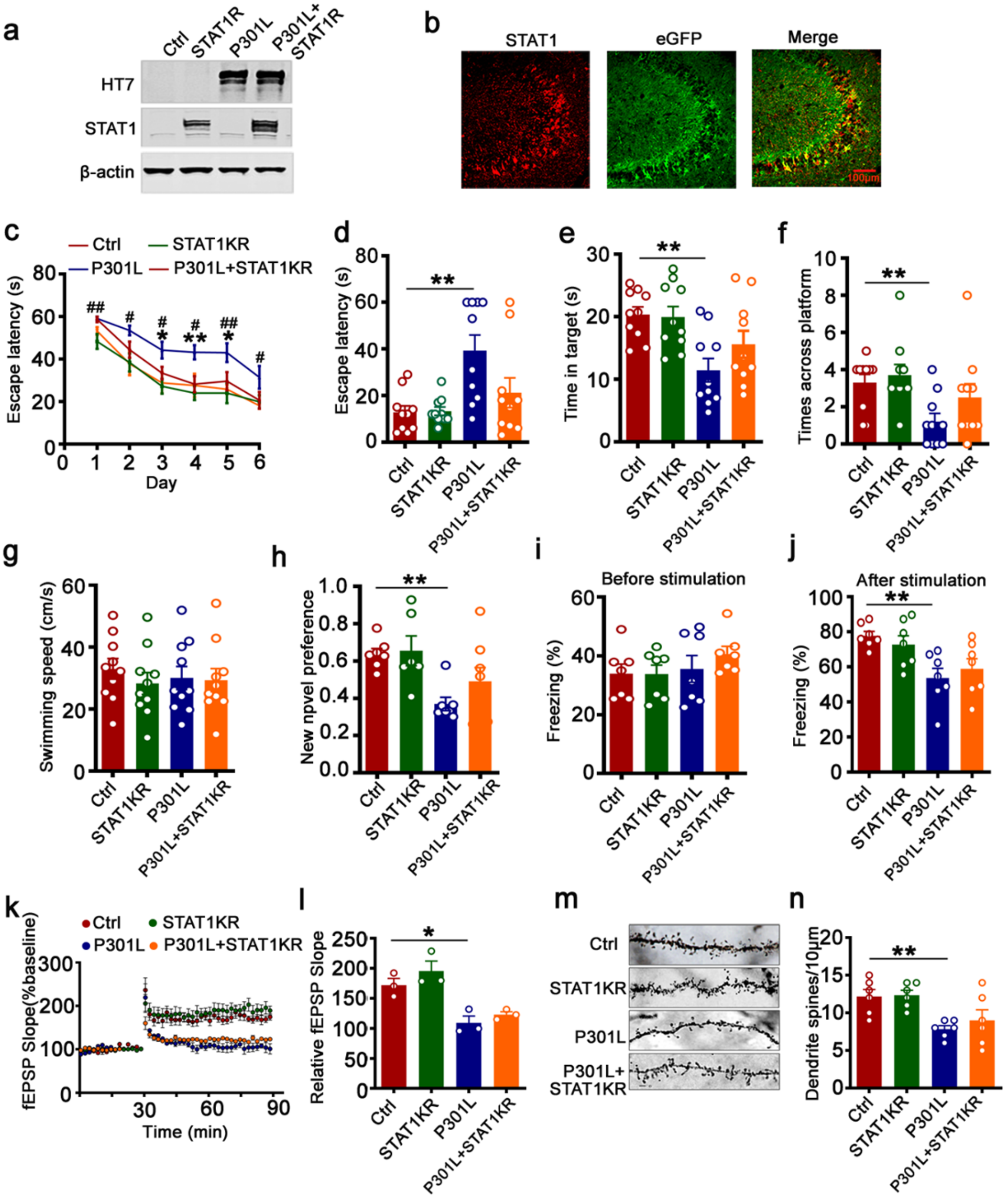

Figure S6 Inhibition of STAT1 acetylation does not ameliorate P301L-hTau-induced synaptic impairment and memory deficits.

AAV-P301L-hTau-eGFP (P301L, 1.3×10^{13} v.g./ml) with AAV-K410/413R-STAT1 (STAT1KR, 1.1×10^{13} v.g./ml) were stereotaxically injected into the hippocampal CA3 of 2-month-old C57 mice. The learning and memory were detected 1 month later.

(a, b) Upregulation of STAT1KR was confirmed by Western blotting and immunofluorescence staining.

(c) Inhibition of STAT1 acetylation ameliorated P301L-induced spatial learning deficits, as shown by the decrease escape latency during MWM training. Two-way repeated measures analysis of variance (ANOVA) followed by Bonferroni's post hoc test, P301L vs Ctrl [day 3] p=0.038, [day 4] p=0.004, [day 5] p=0.022; P301L+STAT1KR vs P301L [day 1] p=0.009; [day 2] p=0.05, [day 3] p=0.018, [day 4] p=0.035, [day 5] p=0.003, [day 6] p=0.031. N =10.

(d-g) Inhibition of STAT1 acetylation did not ameliorate P301L-induced spatial learning deficits as shown by the unaltered escape latency to reach the platform quadrant **(d),** the unaltered time spent in the target quadrant (e), the unaltered times to cross the platform **(f)**, as measured at day 8 by removing the platform. No motor dysfunction was seen **(g)**. Two-way repeated measures analysis of variance (ANOVA) followed by Bonferroni's post hoc test, d, p=0.001; e, p=0.001; f, p=0.0012; n=10.

(h) Inhibition of STAT1 acetylation did not ameliorate P301L-induced cognition impairment, as shown by the lack of variability in time spent in exploring the new novel. Two-way repeated measures analysis of variance (ANOVA) followed by Bonferroni's post hoc test, p=0.004, n=7.

(i, j) Inhibition of STAT1 acetylation did not ameliorate P301L-induced long-term memory deficits, as shown by the invariable freezing time measured by fear conditioning test (n=8-10 each group). Two-way repeated measures analysis of variance (ANOVA) followed by Bonferroni's post hoc test, j, p=0.002, n=7.

(k, l) Inhibition of STAT1 did not restore slopes of field excitatory postsynaptic potential (fEPSP,) recorded in hippocampal CA3, with quantitative analysis. Two-way repeated measures analysis of variance (ANOVA) followed by Bonferroni's post hoc test, p=0.027, n=6~8 slices from 3 mice per group.

(m, n) Golgi staining showed that the density of dendritic spine of the mice treatment with P30L-hTau remained unaltered by overexpression of STAT1-KR. Scale bars, 5μm. Two-way repeated measures analysis of variance (ANOVA) followed by Bonferroni's post hoc test, $p=0.004$, n=at least 20 neurons were analyzed from 6 mice per group.

Data were presented as mean ±SEM.

Figure S7

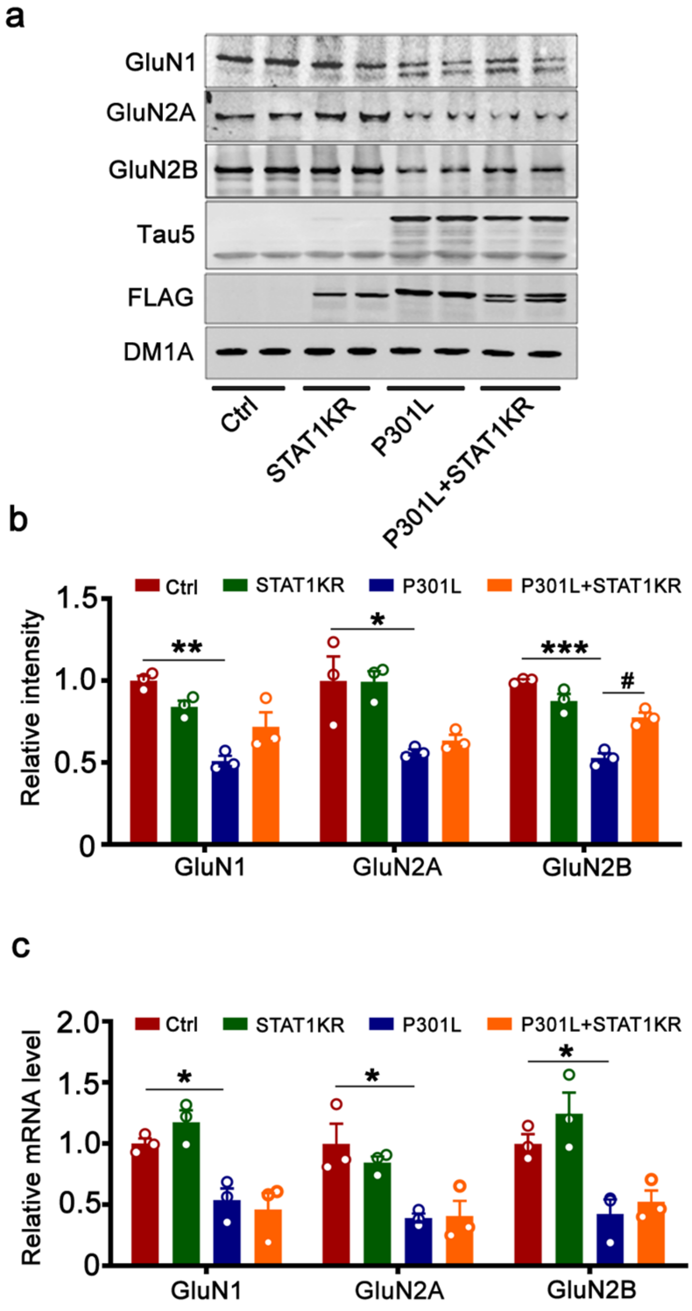

Figure S7 Non-acetylated STAT1 had no effect on the decreased level of NMDARs induced by P301L *in vivo*.

AAV-P301L-hTau-eGFP (P301L, 1.3×10^{13} v.g./ml) alone or with AAV-K410/413R-STAT1 (STAT1KR, 1.1×10^{13} v.g./ml) were stereotaxically injected into hippocampal CA3 of 2-month-old C57 mice. After 1 month, the protein (**a, b**) and mRNA levels (**c**)

of NMDARs were detected by Western blotting and RT-PCR. Two-way analysis of variance (ANOVA) followed by Bonferroni's post hoc test; b, [GluN1] $p=0.001$, [GluN2A] $p=0.024$, [GluN2B] ***$p<0.001$, #$p=0.0138$. c, [GluN1] $p=0.0407$, [GluN2A] $p= 0.0161$, [GluN2B]$p=0.0397$; $n=3$.

Data were presented as mean ± SEM.

Figure S8

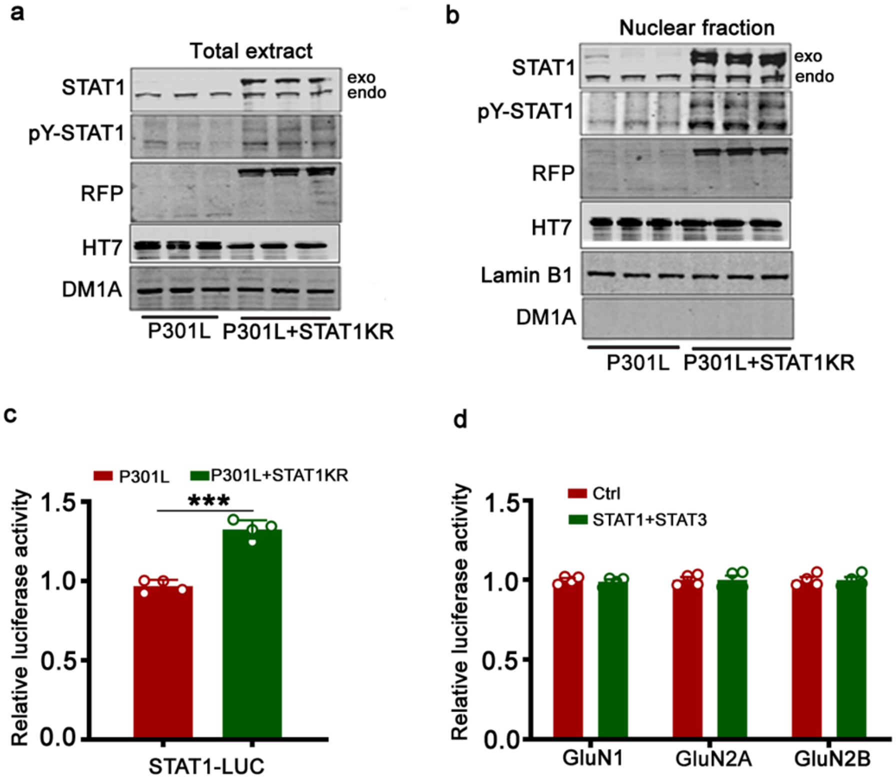

Figure S8 Non-acetylated STAT1 activates STAT1

(**a, b)** Overexpression of RFP-K410/K413R-STAT1 (STAT1KR) plasmid increased endogenous and exogenous STAT1 and pY-STAT1 in the nuclear fraction **(b)** of HEK293 cells with P301L transfection (endo: endogenous; exo: exogenous).

(**c**) Overexpression of RFP-K410/K413R-STAT1 (STAT1KR) plasmid increased luciferase activity of STAT1 in HEK293 cells with P301L transfection. Two-tailed Student's t-test, $p<0.001$, $n=4$.

(d) Overexpression of STAT1 with STAT3 plasmids did not affect the luciferase activity of NMDARs in HEK293 cells.

Data were presented as mean ± SEM.

Figure S9

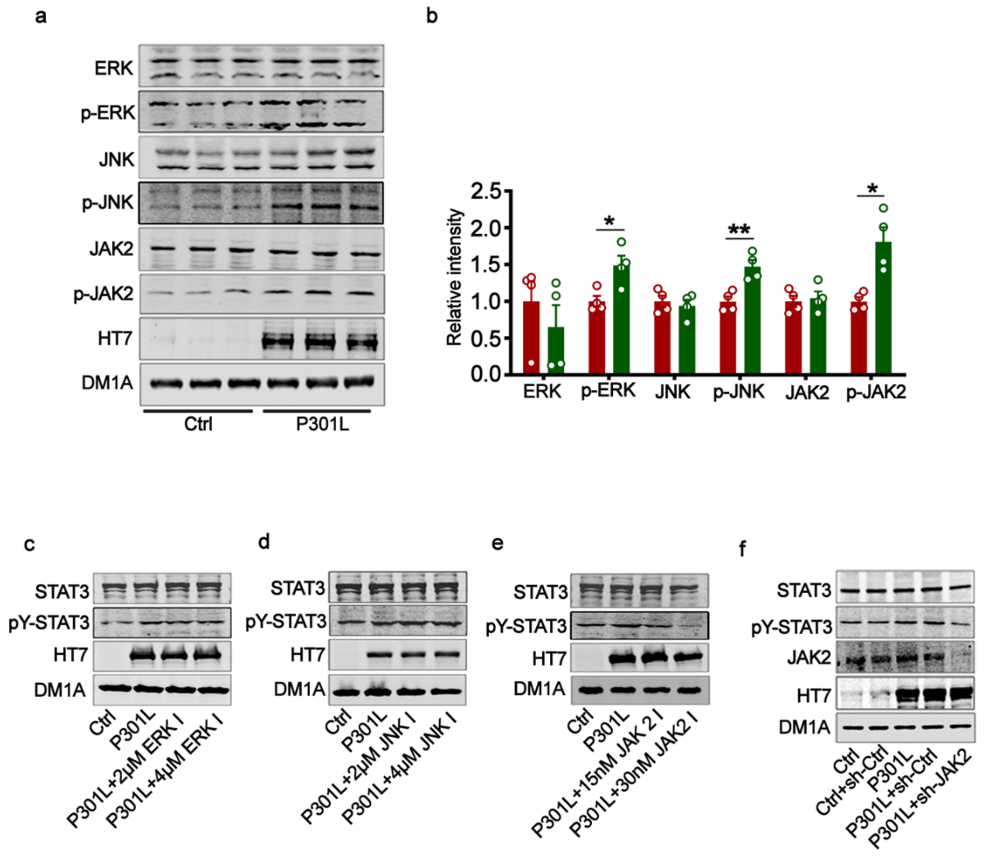

Figure S9 JAK2 activation induced phosphorylation of STAT3 at Tyr705.

(a, b) Overexpression of P301L-hTau in HEK293 cells for 48 h increased the activity-dependent phosphorylation of JAK2, JNK and ERK, as compared with the empty vector control (Ctrl) measured by Western blotting. Two-tailed Student's t-test, [p-ERK] p= 0.0184, [p-JNK]p=0.0056, [p-JAK2]p=0.021, n=4.

(c-f) Pharmacological inhibition of ERK **(c)** or JNK **(d)** for 24 h did not change P301L-hTau-induced STAT3 phosphorylation at Tyr705 in total extracts. Pharmacological inhibition or shRNA transfection of JAK2 (**e, f**) abolished the P301L-hTau-induced STAT3 phosphorylation at Tyr705 in total extracts (n=4).

Figure S10

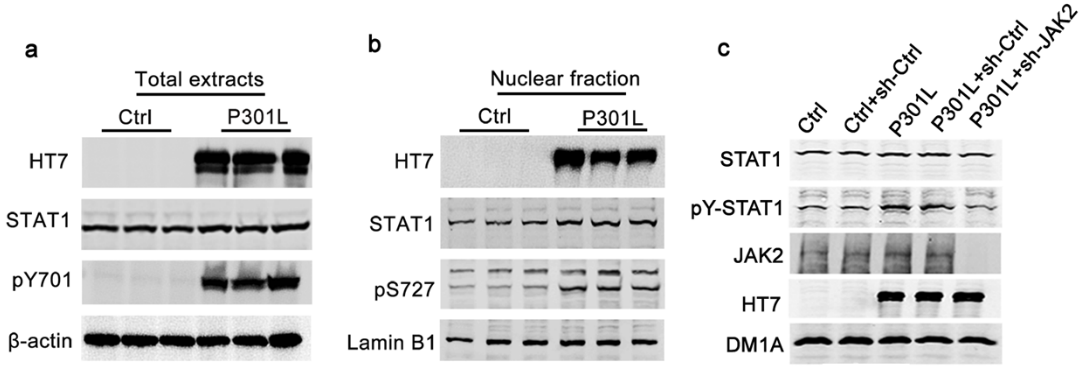

Figure S10 Overexpressing P301L-hTau increased STAT1 nuclear translocation *in vitro*.

Overexpression of P301L-hTau induced the increase total STAT1 level in total extract (a) and phosphorylated STAT1 (pY701) level in the nuclear fraction (b). shRNA transfection of JAK2 (**c**) abolished the P301L-hTau-induced STAT1 phosphorylation at Tyr701 in total extracts (n=4).

Figure S11

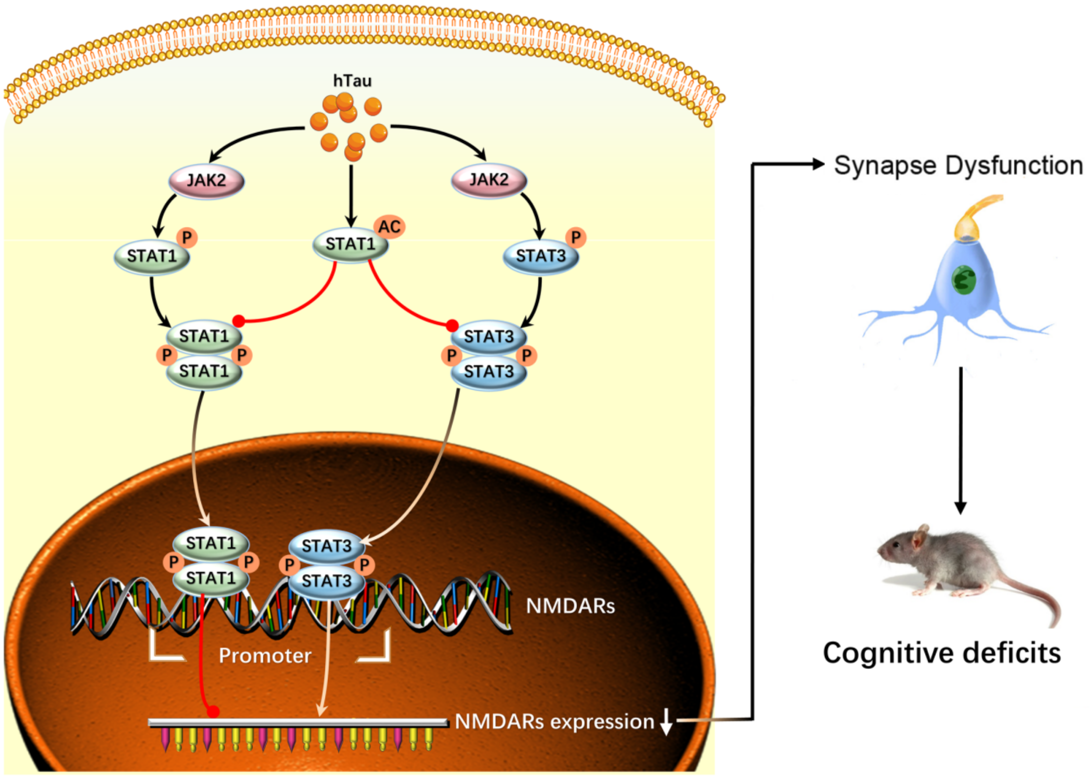

Figure S11 'Trade-off hypothesis' for hTau (P301L) accumulation induced synaptic toxicities.

Tau accumulation activated JAK2/STAT1 signaling pathway to phosphorylate STAT1 at Y701, promoted STAT1 to form a homodimer and translocate to the nucleus, thus activating STAT1 to suppress NMDARs expression and caused cognitive deficits. To rectify the adverse consequences of STAT1 activation, tau acetylated STAT1 to inhibit STAT1 nuclear translocation. However, acetylated STAT1 promoted STAT1 binding with STAT3 to form a heterodimer in the cytoplasm and prevented STAT3 transport into the nucleus, though activated JAK2 also phosphorylated STAT3 at Y705. Tau accumulation inhibited expression of NMDARs through upregulation of STAT1 activity and downregulation of STAT3 activity, ultimately leading to synaptic impairments and cognitive deficits.

Table S1 The antibodies used in the study

Antibody	Specificity	Type	Dilution	Source
STAT3	Total STAT3	Mono-	1:100 for IF<br>1:200 for IHC<br>1:1000 for WB	Cell Signaling(Boston, MA)
STAT3	Total STAT3	Mono-	1:50 for IP	Abcam(Cambridge, UK)
pY-STAT3	p-STAT3 at Tyr 705	Mono-	1:500 for WB	Cell Signaling(Boston, MA)
pS-STAT3	p-STAT3 at Ser 727	Mono-	1:1000 for WB	Cell Signaling(Boston, MA)
STAT1	Total STAT1	Mono-	1:100 for IF<br>1:1000 for WB	Abcam(Cambridge, UK)
STAT1	Total STAT1	Poly-	1:50 for IP	Millpore(Deutschland, Germany)
pY-STAT1	p-STAT1 at Tyr 701	Mono-	1:500 for WB	Cell Signaling(Boston, MA)
pS-STAT1	p-STAT1 at Ser 727	Mono-	1:500 for WB	Cell Signaling(Boston, MA)
Ace- Lysine	acetylated proteins	Mono-	1:1000 for WB	Cell Signaling(Boston, MA)
GluN1	Total NMDAR1	Mono-	1:1000 for WB	Abcam(Cambridge, UK)
GluN2A	NMDAR2A C-term	Poly-	1:1000 for WB	Abcam(Cambridge, UK)
GluN2B	NMDAR2B C-term	Poly-	1:1000 for WB	Abcam(Cambridge, UK)
GluA1	Total GluA1	Mono-	1:500 for WB	Cell Signaling(Boston, MA)
GluA2	Total GluA2	Mono-	1:500 for WB	Cell Signaling(Boston, MA)
SYN	Total Synaptophysin	Poly-	1:1000 for WB	Abcam(Cambridge, UK)
SYT	Total Synaptotagmin	Mono-	1:1000 for WB	Abcam(Cambridge, UK)
CBP	Total CBP	Mono-	1:500 for WB	Cell Signaling(Boston, MA)
P300	Total p300	Mono-	1:1000 for WB	Cell Signaling(Boston, MA)
HDAC2	Total HDAC2	Mono-	1:500 for WB	Abcam(Cambridge, UK)
DM1A	α-Tubulin	Mono-	1:1000 for WB	Abcam(Cambridge, UK)
β-Actin	Human Actin	Poly-	1:2000 for WB	Abcam(Cambridge, UK)
Lamin B1	Nuclear Envelope Marker	Poly-	1:1000 for WB	Abcam(Cambridge, UK)
TAU5	Total tau	Mono-	1:1000 for WB	Abcam(Cambridge, UK)
FLAG	FLAG-tag	Mono-	1:1000 for WB	Abcam(Cambridge, UK)
GFP	GFP-tag	Mono-	1:1000 for WB	Abcam(Cambridge, UK)
RFP	RFP-tag	Poly-	1:1000 for WB	Abcam(Cambridge, UK)
HT7	Total human tau	Mono-	1:1000 for WB<br>1:200 for IF	Thermo Fisher(Waltham, MA)
AT8	Human PHF-tau	Mono-	1:200 for IF	Thermo Fisher(Waltham, MA)
TDP43	Total TDP43	Poly-	1:1000 for WB	Proteintech(Chicago, Illinois)
α-synuclein	Total α-synuclein	Poly-	1:1000 for WB	Proteintech(Chicago, Illinois)
GFAP	Total GFAP	Mono-	1:200 for IF	Cell Signaling(Boston, MA)
IBA1	Total IBA1	Mono-	1:200 for IF	Wako(JAPAN)
NeuN	Total NeuN	Mono-	1:200 for IF	Cell Signaling(Boston, MA)
pS214	p-Tau at Ser214	Poly-	1:1000 for WB	Signalway Antibody(College Park, Maryland)
pT231	p-Tau at Thr231	Poly-	1:1000 for WB	Signalway Antibody(College Park, Maryland)
pS396	p-Tau at Ser396	Poly-	1:1000 for WB	Signalway Antibody(College Park, Maryland)
pS404	p-Tau at Ser404	Poly-	1:1000 for WB	Signalway Antibody(College Park, Maryland)
ERK	Total ERK	Poly-	1:1000 for WB	Cell Signaling(Boston, MA)

p-ERK	p-ERK at Thr202/Thr204	Poly-	1500 for WB	Cell Signaling(Boston, MA)
JNK	Total JNK	Poly-	1:300 for WB	Santa Cruz Technology(Dallas, TX)
p-JNK	p-ERK at Tyr185	Poly-	1:500 for WB	Cell Signaling(Boston, MA)
JAK2	Total JAK2	Poly-	1:1000 for WB	Cell Signaling(Boston, MA)
p-JAK2	p-JAK2 at Tyr1007/1008	Poly-	1:500 for WB	Cell Signaling(Boston, MA)

Table S2 Human brain tissues used in the study

Case Number	Primary Neuropathologic Diagnosis	Secondary Neuropathologic Diagnosis	Formalin Tissue	PMI (hr)	Age at Death	ApoE	Race/ Sex
E08-101	Control	Large cerebral hemorrhage	√	11.5	78	E3/3	wf
E11-33	Control	Microinfarct-pR	√	15	43	E3/3	bf
OS98-06	FTDP-17 (P301L)	Infarcts	√	5	63	E3/3	wm
OS95-38	FTDP-17 (P301L)	PD	√	5	55	E3/3	wf
